# Synthesis and Unusual Reactivity of Acyl-Substituted 1,4-Disilacyclohexa-2,5-dienes

**DOI:** 10.1021/acs.organomet.2c00475

**Published:** 2022-11-14

**Authors:** Lukas Schuh, Ana Torvisco, Michaela Flock, Christa Grogger, Harald Stueger

**Affiliations:** Institute of Inorganic Chemistry, Graz University of Technology, Stremayrgasse 9, Graz 8010, Austria

## Abstract

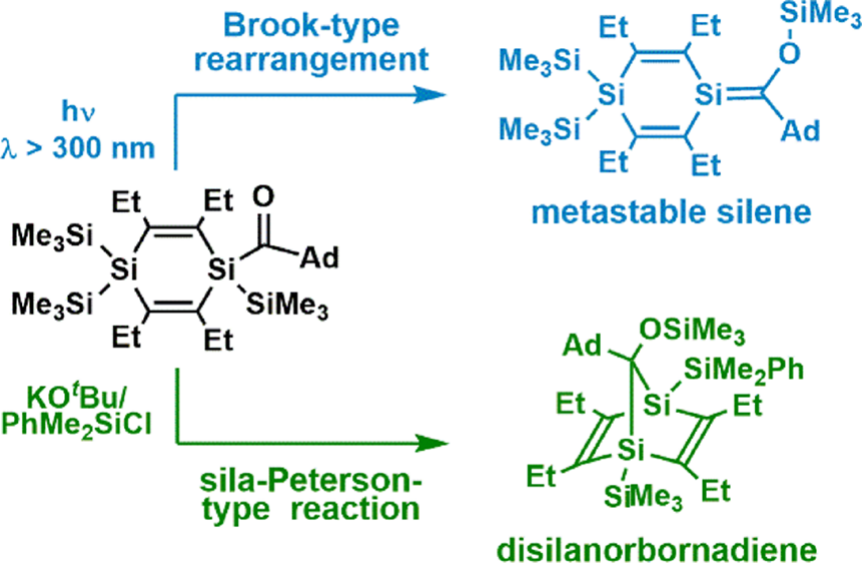

In continuation of our recent studies on group 14 rings with exocyclic silicon–carbon double bonds, we report here on the synthesis and reactivity of previously unknown acyl-substituted 1,4-disilacyclohexa-2,5-dienes. 1,1,4,4-Tetrakistrimethylsilyl-1,4-disilacyclohexa-2,5-diene **1** cleanly afforded the silyl anion **1-K** after addition of 1 equiv of KO^*t*^Bu. **1-K** subsequently could be reacted with various electrophiles to the expected substitution products including compounds **4** and **5**. When photolyzed with λ > 300 nm radiation, **4** and **5** undergo Brook-type 1,3-Si → O migration reactions to generate the corresponding 1,4-disilacyclohexadienes with exocyclic Si=C bonds as the primary products. These metastable silenes only could be characterized in form of appropriate quenching products. The reaction of compound **4** with KO^*t*^Bu followed by the addition of 1 equiv of PhMe_2_SiCl surprisingly gave the silylated 1,4-disilanorbornadiene cages **8** and **9** instead of the expected exocyclic silene. The responsible sila-Peterson-type mechanism could be elucidated by density functional theory calculations at the conductor-like polarizable continuum model (THF) B3LYP-GD3/6-31 + G(d) level and by the isolation and characterization of unstable intermediate products after proper derivatization.

## Introduction

π-Conjugated organic rings incorporating silicon atoms recently attracted great attention due to their outstanding electronic nature and potential applications in electronic devices. Siloles (silacyclopenta-2,4-dienes) are the most thoroughly studied representatives of this class of compounds because they are readily prepared on a preparative scale with a wide range of substitution patterns.^1−6^ High-efficiency light-emitting diodes, organic solar cells, and high-mobility field-effect transistors have all been reported based on silole derivatives, indicating the potential importance of siloles to materials chemistry. Related studies involving six-membered 1,4-disilacyclohexadienes were more or less restricted to synthetic issues^7−14^ until Ottosson and co-workers discovered that this class of compounds may complement or even outperform siloles in the design of electronically active materials.^15−17^ In particular for 1,1,4,4-tetrakistrimethylsilyl-1,4-disilacyclohexa-2,5-diene **1**, strong cyclic cross-hyperconjugation was detected as shown by its long wavelength UV absorption and its unusually low oxidation potential and valence photoionization energies.

**Chart 1:**
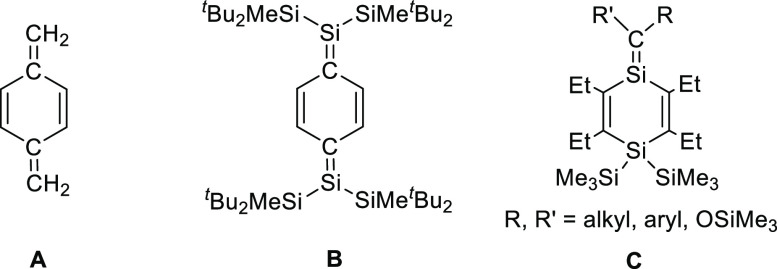
Structures of *p*-Quinodimethane **A** and the Related Si-Containing Derivatives **B** and **C**

The electronic structure of 1,4-disilacyclohexa-2,5-dienes is strongly reminiscent of the π-conjugated *p*-quinodimethanes (*p*-QDM) which are currently investigated intensively with respect to modern applications in organic field-effect transistor or organic photovoltaic devices.^18,19^ The parent *p*-QDM **A** (Chart 1), however, is highly reactive due to its energetically low-lying benzenoid diradical transition state^20^ and readily polymerizes even at low temperatures in the absence of electron-withdrawing exocyclic X-groups or steric protection which limits the scope of possible applications.

In 2011, Sekiguchi and co-workers reported the isolation and structural characterization of the *p*-disilaquinodimethane **B** which is stabilized kinetically by bulky silyl groups at the exocyclic Si-atoms.^21^ Reactivity studies performed for **B** revealed significant contribution of the singlet diradical resonance form to the ground electronic state. In this context, 1,4-disilacyclohexadienes with exocyclic Si=C double bonds such as compound **C** are interesting synthetic targets because they represent excellent models to study cyclic cross-conjugational-type interactions between the endocyclic C=C– and the exocyclic Si=C double bonds via the bridging silicon atom.

Recently, a series of unprecedented stable cyclohexasilanes that contain exocyclic Si=C double bonds were synthesized successfully in our laboratories.^22−26^ Based on the synthesis concepts developed in these studies, this paper describes the preparation and structural identification of the first 1,4-disilacyclohexa-2,5-dienyl anion **1-K** and its reaction with various electrophiles. Additionally, we present the outcome of our attempts to convert the newly synthesized acyl-1,4-disilacyclohexa-2,5-dienes **4** and **5** to the corresponding type **C** silenes along with computational studies on the underlying reaction mechanism.

## Results and Discussion

### Synthesis of 1-Potassio-1,4,4-tristrimethylsilyl-1,4-disilacyclohexa-2,5-diene

**Scheme 1:**
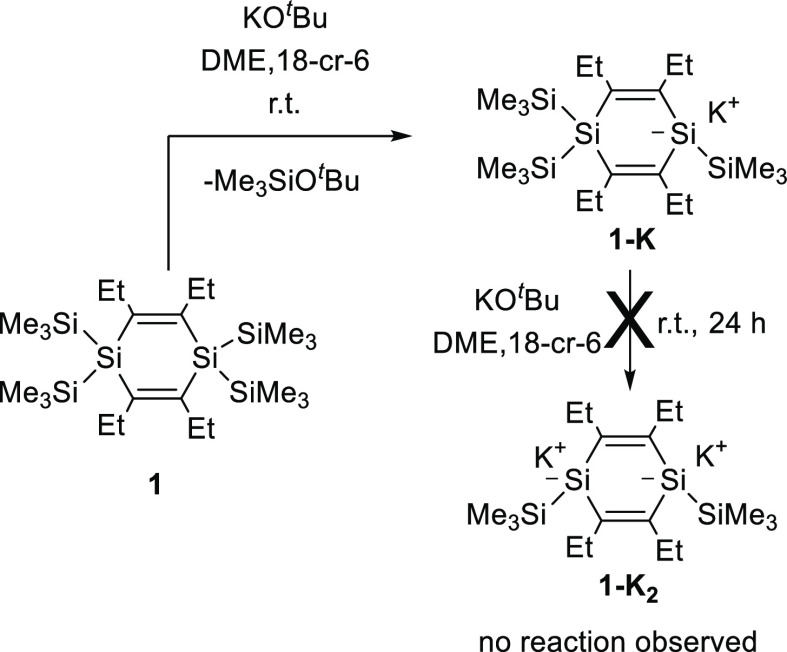
Reaction of **1** with KO^*t*^Bu

As shown in Scheme 1, 1,1,4,4-tetrakistrimethylsilyl-1,4-disilacyclohexa-2,5-diene **1** cleanly reacts with 1 equiv of KO^*t*^Bu in the presence of 18-cr-6 to the silyl anion **1-K**. According to nuclear magnetic resonance (NMR) analysis, **1-K** is formed without any detectable byproducts and can be isolated as the 18-cr-6 adduct in ∼90% yield as a red-brown semisolid residue after removal of the solvent in vacuo. **1-K** was characterized by multinuclear NMR spectroscopy. ^1^H-, ^13^C-, and ^29^Si data are summarized in the Experimental Section, and the experimental spectra are depicted in Figures S1–S3. Unexpectedly, the NMR data of **1-K** suggest a highly symmetric structure. In particular, all of the SiMe_3_ groups, both endocyclic silicons and all ring carbon atoms, were observed to be equivalent. This may arise from an unprecedented 1,4-shift of a SiMe_3_ group within the NMR time scale. This explanation seems conclusive although VT-NMR experiments performed down to −40 °C showed only insignificant changes in the ^1^H NMR spectra. VT NMR measurements at even lower temperatures or VT ^29^Si- or ^13^C NMR studies were impossible due to the rapidly decreasing solubility of **1**-**K**.

**Figure 1:**
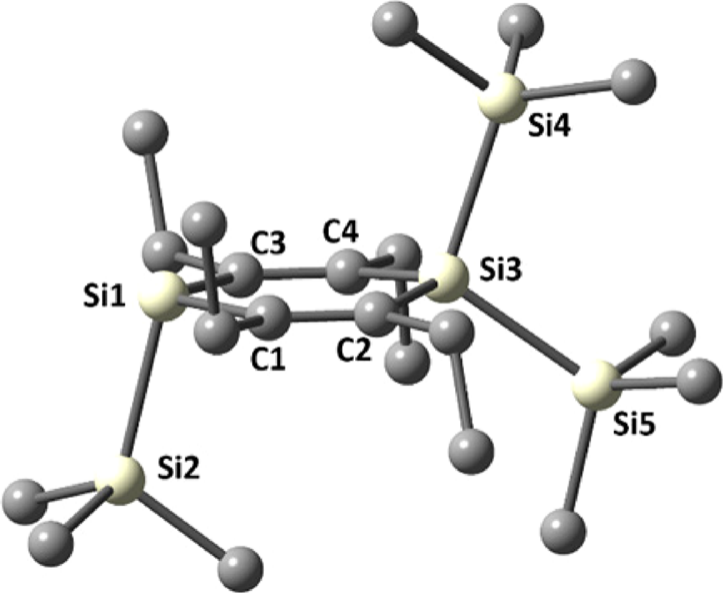
Density functional theory conductor-like polarizable continuum model (DFT CPCM) (THF) B3LYP-GD3/6-31 + G(d) calculated structure of the most stable conformer of **1-K**. All hydrogen atoms are omitted for clarity. Selected calculated bond lengths [pm] and bond and torsional angles [deg]: Si(1)–Si(2) 238.9, Si(3)–Si(4) 238.5, Si(3)–Si(5) 238.8, Si–C_endo_ (mean) 191.0, C(1)–C(2) 136.4, C(3)–C(4) 136.4; Si(1)–C(1)–C(2) 127.5, C(1)–C(2)-Si(3) 122.1, C(2)–Si(3)–C(4) 113.0, Si(3)–C(4)–C(3) 121.8, C(4)–C(3)–Si(1) 127.6, C(3)–Si(1)–C(1) 107.1; Si(1)–C(1)–C(2)–Si(3) 3.9, C(1)–C(2)–Si(3)–C(4) −10.3, C(2)–Si(3)–C(4)–C(3) 10.9, Si(3)–C(4)–C(3)–Si(1) −5.0, C(4)–C(3)–Si(1)–C(1) −2.0, C(3)–Si(1)–C(1)–C(2) 2.6.

Although numerous attempts were made, crystals of **1-K** suitable for X-ray crystallography could not be obtained. Not surprising, in the most stable DFT CPCM (THF) B3LYP-GD3/6-31 + G(d) calculated anion structure (Figure 1), the ethyl groups adjacent to the negatively charged silicon atom are arranged at the opposite side of the ring plane relative to the SiMe_3_ group. The calculated ring system is not entirely planar with the Si(SiMe_3_)_2_ group approximately 5° out of the plane. Other conformers with different orientations of the ethyl groups are at least 14.8 kJ/mol higher in energy (Figure S35).

Solutions of **1-K** turned out to be stable at ambient temperature in the absence of air for at least a week. Surprisingly, **1-K** did not react readily to the dianion **1-K_2_** with a second equivalent of KO^*t*^Bu. After stirring an equimolar mixture of **1-K**, KO^*t*^Bu, and 18-cr-6 in dimethoxyethane (DME) for 24 h at 25 °C, no reaction was detected by NMR spectroscopy. Significantly longer reaction times or elevated temperatures resulted in the gradual decomposition of **1-K** to an undefined material. This finding is highly unexpected and contradicts the behavior of 1,1,4,4-tetrakistrimethylsilylcyclohexasilane, which straightforwardly afforded the 1,4-dipotassiodisilanide upon treatment with 2 equiv of KO^*t*^Bu.^27^ Free reaction enthalpies for both reaction steps omitting the potassium counterion were calculated at the CPCM (THF) B3LYP-GD3/6-31 + G(d) level. In agreement with the experiment, the first step, formation of **1-K**, is exergonic (ΔΔ*G* = −50.4 kJ/mol), while the subsequent reaction to the dianion, **1-K_2_**, is endergonic (ΔΔ*G* = +20.0 kJ/mol).

### Reaction of **1-K** with Electrophiles

**Scheme 2:**
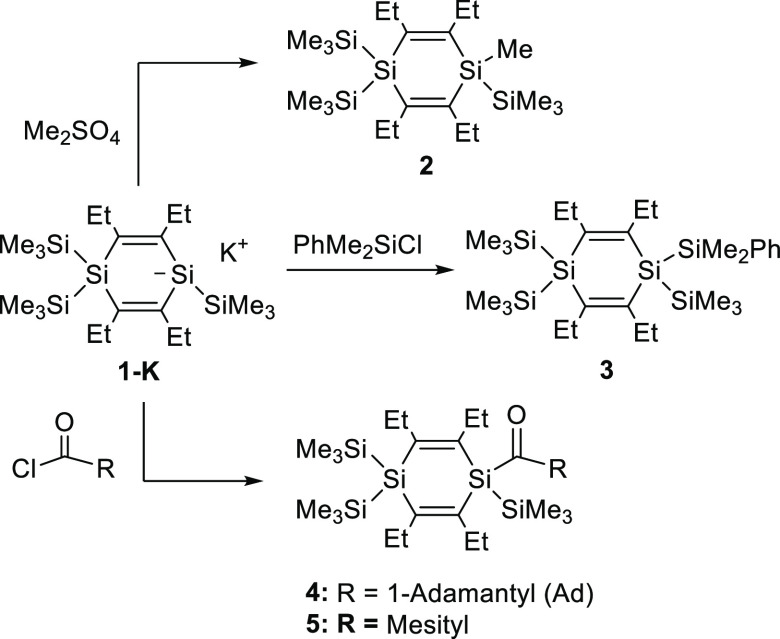
Reactivity of **1-K** versus Electrophiles

**1-K** exhibits the reactivity of a typical silanide anion (Scheme 2). Addition of a DME solution of **1-K** to Me_2_SO_4_ or PhMe_2_SiCl, respectively, gave the expected substitution products **2** and **3**, which were isolated as colorless crystals in good yield after crystallization from acetone at −30 °C. The reaction of **1-K** with acid chlorides proceeded analogously and enabled the synthesis and isolation of the previously unknown acyl-1,4-disilacyclohexa-2,5-dienes **4** and **5**.

Analytical and spectroscopic data obtained for **2**–**5** (for details consult the Experimental Section) are consistent with the proposed structures. NMR chemical shift values are close to the ones observed for compound **1**. However, substitution of one SiMe_3_ group in **1** produces two sets of magnetically nonequivalent endocyclic carbon atoms and ethyl groups. The resulting number of resonance lines actually appears in the experimental ^1^H- and ^13^C-NMR spectra, although some ^1^H signals are too close to each other to be completely resolved. In accordance with the asymmetry of the molecules, ^29^Si-NMR spectra of compounds **2**, **4**, and **5** exhibit three, and compound **3** exhibits four, resonances between −16 and −21 ppm for the exocyclic SiR_3_ groups and two signals for the nonequivalent endocyclic silicons between −31.6 and −53.3 ppm. As expected, the carbon substituents at one endocyclic silicon atom in **2**, **4**, and **5** provoke a significant high field shift of the corresponding ^29^Si resonance by at least 10 ppm relative to compound **1**.^28^ DFT calculations of the ^29^Si chemical shifts show a similar picture. ^29^Si chemical shifts of the endocyclic Si atoms are computed at −53.6 ppm/–31.6 ppm for **2**, −59.1 ppm/–49.7 ppm for **4**, and −54.7 ppm/–42.6 ppm for **5** in good agreement with measured values.

**Figure 2:**
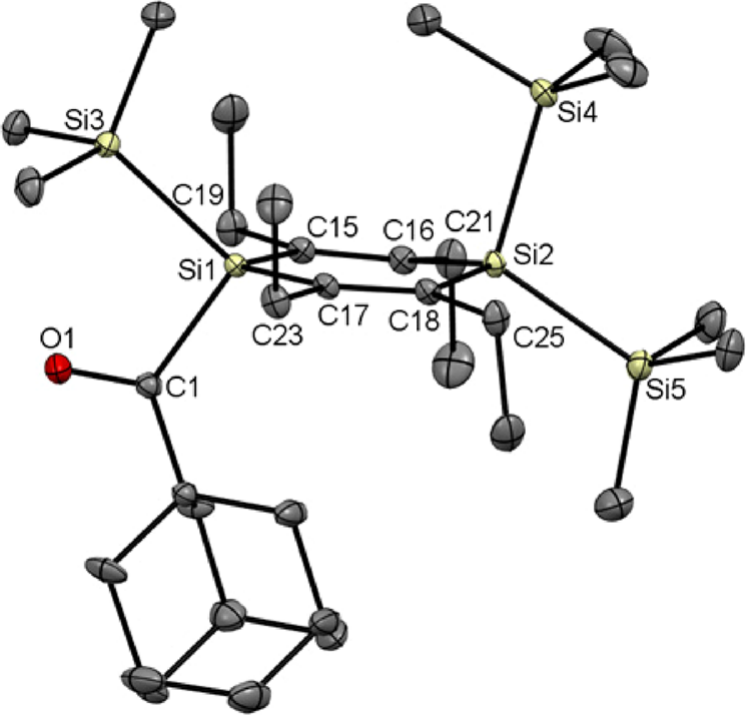
Molecular structure of **4**. All hydrogen atoms are omitted for clarity. Thermal ellipsoids are set at the 30% probability level. Selected bond lengths [pm] and bond and torsional angles [deg] with estimated standard deviations: Si–Si (mean) 237.9, Si(1)–C(1) 1.971(1), Si–C_endo_ (mean) 188.2, C(15)–C(16) 135.7(1), C(17)–C(18) 135.5(1), C(1)–O(1) 122.4(1); Si(1)–C(15)–C(16) 123.2(1), C(15)–C(16)–Si(2) 123.5(1), C(16)–Si(2)–C(18) 111.5(1), Si(2)–C(18)–C(17) 122.8(1), C(18)–C(17)–Si(1) 124.4(1), C(17)–Si(1)–C(15) 112.4(1); Si(1)–C(17)–C(18)–Si(2) 7.6(1), C(17)–C(18)–Si(2)–C(16) −14.2(1), C(18)–Si(2)–C(16)–C(15) 17.1(1), Si(2)–C(16)–C(15)–Si(1) −12.8(1), C(16)–C(15)–Si(1)–C(18) 4.0(1), C(15)–Si(1)–C(17)–C(18) −1.2(1).

**Figure 3:**
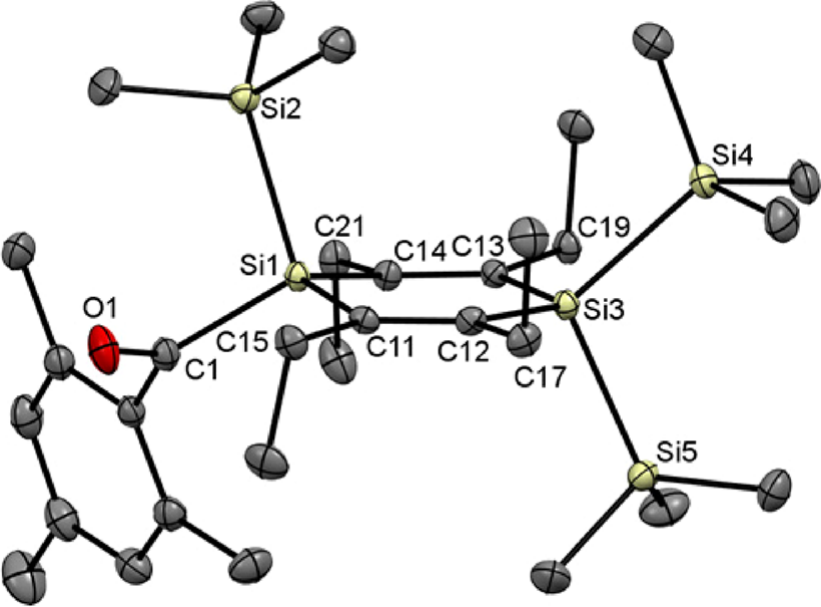
Molecular structure of **5**. All hydrogen atoms are omitted for clarity. Thermal ellipsoids are set at the 30% probability level. Selected bond lengths [pm] and bond and torsional angles [deg] with estimated standard deviations: Si–Si (mean) 237.3, Si(1)–C(1) 194.6(2), Si–C_endo_ (mean) 188.0, C(11)–C(12) 135.2(2), C(13)–C(14) 135.7(2), C(1)–O(1) 122.5(2); Si(1)–C(11)–C(12) 121.8(1), C(11)–C(12)–Si(3) 124.1(1), C(12)–Si(3)–C(13) 111.9(1), Si(3)–C(13)–C(14) 124.0(1), C(13)–C(14)–Si(1) 121.4(1), C(14)–Si(1)–C(11) 113.7(1); Si(1)–C(11)–C(12)–Si(3) −11.9(2), C(11)–C(12)–Si(3)–C(13) 5.5(2), C(12)–Si(3)–C(13)–C(14) −7.4(2), Si(3)–C(13)–C(14)–Si(1) 15.2(2), C(13)–C(14)–Si(1)–C(11) −19.3(1), C(14)–Si(1)–C(11)–C(12) 17.5(1).

Figures 2 and 3 show the crystal structures of the acyl-1,4-disilacyclohexa-2,5-dienes **4** and **5** together with selected bond lengths, bond angles, and dihedral angles. The molecular structures of compounds **2** and **3** showing similar characteristics are discussed in the Supporting Information (Figures S33 and S34). Structural features observed for compounds **4** and **5** compare well with other 1,4-disilacyclohexadiene structures reported in the literature.^9,13,16,29−31^**4** and **5** crystallize in the monoclinic space group *P*21/*n* and in the orthorhombic space group *Pbcn*, respectively. In both structures, the disilacyclohexadiene ring adopts a slightly twisted chair conformation with two ethyl groups above and two ethyl groups below the least-squares plane through the olefinic carbons. The endocyclic Si atoms deviate from the olefinic carbon atom plane by 24.0 and 4.0 pm in **4** and by 27.5 and 9.9 pm in **5**, respectively. This deviation is close to the corresponding value of 20.0 pm observed for compound **1**.^16^ B3LYP/6-31G(d) calculations recently published for compound **1** by Ottosson and co-workers^17^ showed that the nonplanar structure of **1** is primarily caused by steric factors. In line with this picture, the endocyclic SiR_2_ moieties in the structures of **4** and **5** are forced out of planarity in order to minimize unfavorable steric interactions between the bulky R groups at silicon and the adjacent ethyl carbons.

Bond lengths and angles apparent in the structures of **4** and **5** are also unexceptional and closely resemble the corresponding values measured for compound **1**. Endocyclic C=C (135.6 pm for **4** and 135.5 pm for **5**) and mean Si–C bond lengths (188.2 pm for **4** and 188.0 pm for **5**) are moderately larger than normal C=C (133 pm)^32^ and Si–C (186 pm)^33^ bond distances which is common for 1,4-disila-2,5-cyclohexadienes. The structures of the B3LYP-GD3/6-31 + G(d) calculated global minima of **4** and **5** coincide with the solid-state structures with the ring in a slightly twisted chair conformation (Figures S36 and S37). All calculated bond lengths are slightly shorter than in the experimental solid-state structures.

### Photolysis of **4**

**Scheme 3:**
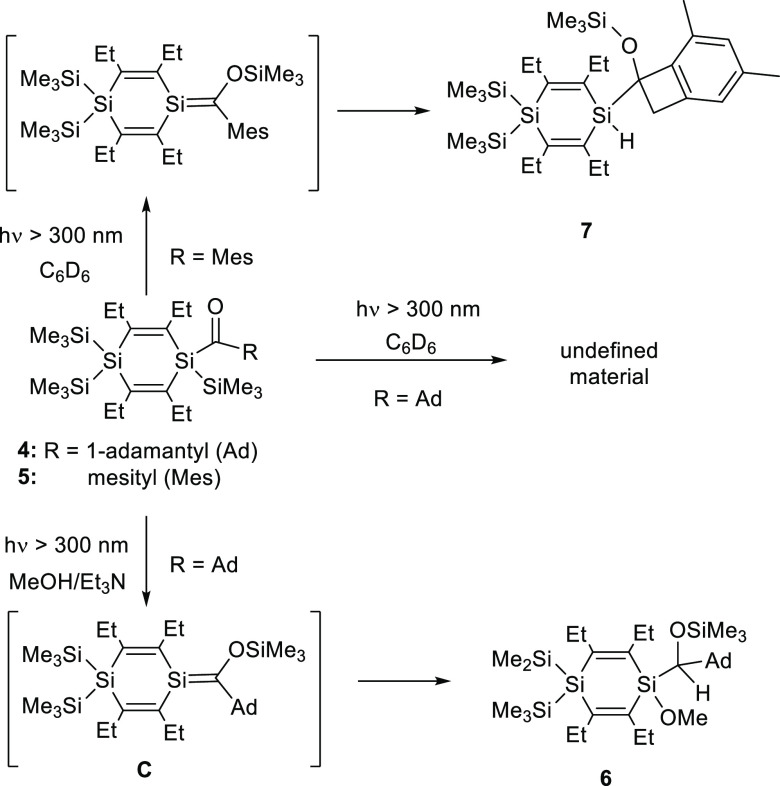
Photolysis of Acyl-1,4-disilacyclohexa-2,5-dienes **4** and **5**

Already in 1981, Brook and coworkers synthesized the first stable species with a Si=C double bond by the photochemically induced 1,3-Si → O shift of a SiMe_3_ group within the acylpolysilane (Me_3_Si)_3_SiC(*O*)Ad.^34^ More recently, we reported the successful preparation of cyclopolysilanes with exo- and endocyclic Si=C double bonds utilizing related photochemical transformations of various acylcyclopolysilanes.^24−26^ Encouraged by these promising results, we now investigated the photolysis of the acyl-1,4-disilahexadiene **4** with the primary aim to generate the corresponding silenes of type **C**. The obtained results are summarized in Scheme 3.

Irradiation of **4** in neat *d*_6_-benzene solution (*c* ≈ 0.25 M) with λ > 300 nm light only afforded a complex mixture of an unidentified material with the unreacted educt. NMR analysis of the reaction solution performed after 30 and 90 min showed the appearance of numerous additional broad lines of unknown origin (Figures S16 and S17). Low-field shifted ^13^C resonance lines characteristic of Brook-type silenes around 210 ppm^35^ could not be detected. Attempts to isolate individual products from this mixture by crystallization or chromatography failed.

Brook-type silenes are very efficiently trapped by alcohols.^35^ When **4** dissolved in *d*6-benzene was photolyzed with λ > 300 nm light for 60 min in the presence of MeOH, to which a trace amount of Et_3_N had been added, the expected 1,2-addition product of the Si=C double bond (compound **6**) was actually obtained.^36^ This finding is certainly of interest because it clearly demonstrates that the photolysis of acyl-1,4-disilacyclohexadienes actually proceeds under the initial formation of the corresponding type **C** silene. Apparently, these silenes are not stable under the photolytical conditions applied in the absence of appropriate trapping agents and decompose to an unidentified material.

Small amounts of pure **6** could be isolated as a colorless oil from the crude photolysis solution by preparative thin-layer chromatography and characterized by NMR spectroscopy and gas chromatography–mass spectrometry (GC–MS) analysis. In line with the structure of **6**, the ^29^Si spectrum exhibits five signals with two resonances at −15.9 and −16.6 ppm for the SiMe_3_ groups, two signals for the endocyclic Si atoms at −20.1 and −55.9 ppm, and one signal for the OSiMe_3_ group at +15.1 ppm. ^13^C- and ^1^H NMR data are also consistent with the proposed structure. GC–MS showed one peak in the gas chromatogram which corresponds to compound **6** according to its mass spectrum. Details are summarized in the Experimental Section; experimental spectra of crude and purified **6** are included in the Supporting Information.

### Photolysis of **5**

Mesityl-substituted Brook-type silenes were shown earlier to undergo photochemically induced addition reactions of a mesityl *ortho*-CH_3_ group to the Si=C double bond under formation of substituted benzocyclobutenes.^37,38^  Compound **5** reacts accordingly and afforded the spirobenzocyclobutene **7** after photolysis in *d*_6_-benzene solution with λ > 300 nm light for 30 min in high selectivity (Scheme 3). Pure **7** could be isolated by preparative thin-layer chromatography in 65% yield and fully characterized. Typical NMR signals for the SiH group [δ(^1^H) = 4.99 ppm; δ(^29^Si) = −43.5 ppm] and for the cyclobutene C-atoms appear [δ(^13^C) = 75.4 ppm (Si*C*O); 41.5 ppm (aryl-CH_2_)].^39^ Otherwise, spectroscopic data are unexceptional. The formation of **7** can be taken as another proof for the involvement of type **C** silenes in the photochemistry of acyl-1,4-disilacyclohexadienes.

### Reaction of **4** with KO^*t*^Bu/R_3_SiCl

Recently, we discovered that 1-acyl-1,4,4-tris(trimethylsilyl)permethylcyclohexasilanes react with KO^*t*^Bu under the clean formation of the corresponding silenolates. Furthermore, we observed that the reactivity of these cyclic silenolates versus chlorosilanes depends on the substituents attached to the carbonyl C-atom.^23^ While alkyl-substituted silenolates smoothly reacted with an equimolar amount of R_3_SiCl to give the Si-silylated acylcyclohexasilane, the aryl-substituted compounds under the same conditions exclusively afforded the Brook-type silene. This finding parallels the chemical behavior of Ohshita’s and Ishikawa’s lithium silenolates^40^ and was explained by the different coordination of the K^+^ counterion to the SiC(O)R moiety and the resulting increased enol character of the aryl-substituted silenolates.

**Scheme 4:**
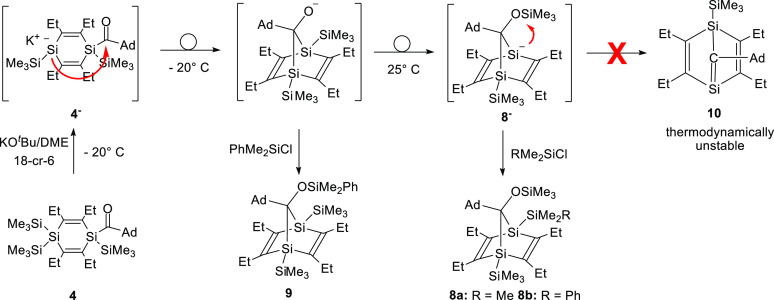
Intramolecular Sila-Peterson Reaction of Acyl-1,4-disilacyclohexa-2,5-diene **4**

In disagreement with these observations, the 1,4-disilanorbornadienes **8a**,**b** were isolated in >60% yield as the sole reaction products, after compound **4** had been treated with 1 equiv of KO^*t*^Bu/18-cr-6 at −20 °C followed by warming to room temperature and addition of the resulting red solution to 1.1 equiv of Me_3_SiCl or PhMe_2_SiCl, respectively (Scheme 4). If KO^*t*^Bu was added at −20 °C and the reaction mixture was not allowed to warm to room temperature prior to the addition of PhMe_2_SiCl, exclusively the norbornadiene **9** with the PhMe_2_Si group in the apical position was formed.

**Scheme 5:**
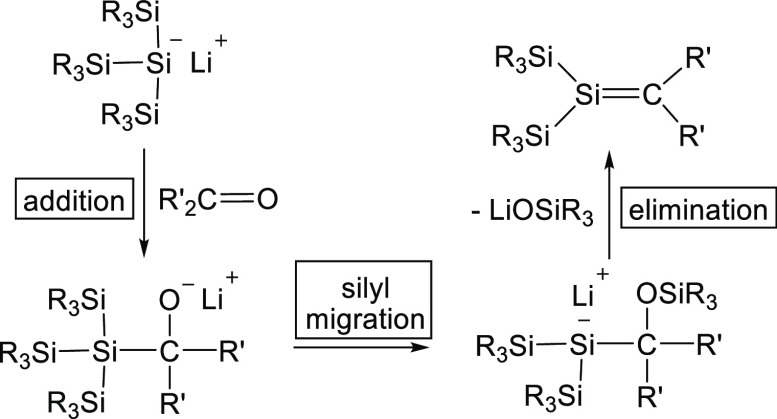
General Scheme of the Sila-Peterson Reaction

From the observed product distribution, it is obvious that the reaction of **4** with KO^*t*^Bu follows an intramolecular sila-Peterson-type mechanism. The sila-Peterson reaction (Scheme 5) was discovered independently by the groups of Oehme,^41,42^ and Ishikawa^43^ and nowadays belongs to the standard procedures suitable for the synthesis of stable or metastable silenes.^44−49^

The key step is the 1,2-elimination of a silanolate from an α-siloxypolysilane, obtained by the interaction of a polysilyllithium compound with an aldehyde or ketone under formation of a silene. In the case of **4**, this final step would result in the formation of the highly strained bicyclic silene **10** which apparently is not formed. DFT calculations performed at the CPCM (THF) B3LYP-GD3/6-31 + G(d) level of theory also showed that **10** is not a thermodynamically stable compound. Consequently, solutions of the 1,4-disilanorbornadienide anion **8^–^** are remarkably stable at room temperature under inert gas. Addition of a DME solution of **8^–^** which had been stored before for 1 week at 25 °C to PhMe_2_SiCl still afforded the expected quenching product **8b** without any noticeable byproducts.

The structures of compounds **8a**,**b** and **9** were confirmed by multinuclear NMR spectroscopy, elemental analysis, and mass spectrometry.^50^ Compounds **8a** and **9** have two sets of two chemically equivalent carbons, while in **8b**, the endocyclic unsaturated carbons are all chemically nonequivalent. The respective number of resonance lines actually appears in the ^13^C NMR spectra. It is interesting to note that the ^13^C signals of the C=C groups of compounds **8a**,**b** and **9** are shifted to a lower field by about 10 ppm relative to comparable 1,4-disilacyclohexadienes such as compounds **1** or **2**. Similar low-field shifts are apparent if one compares fully carbon-based 1,4-cyclohexadienes and appropriately substituted norbornadienes.^51,52^

**Figure 4:**
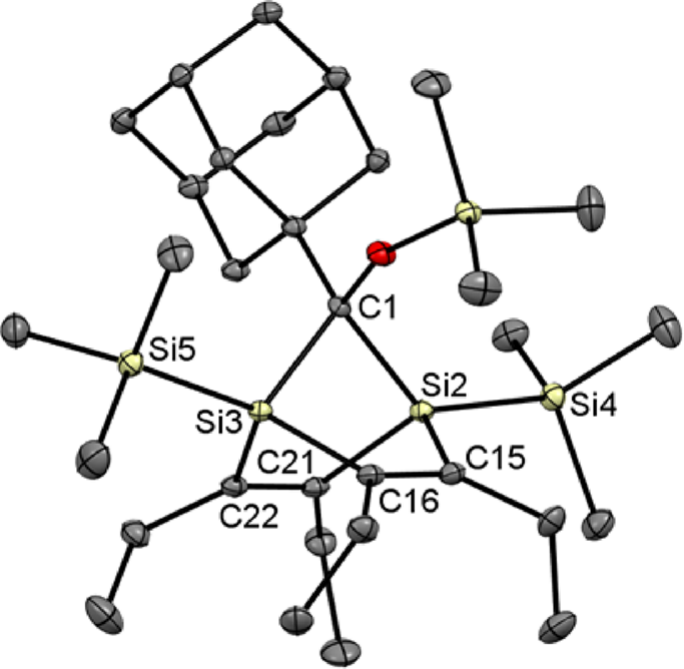
Molecular structure of **8a**. All hydrogen atoms are omitted for clarity. Thermal ellipsoids are set at the 30% probability level. Selected bond lengths [pm] and bond and torsional angles [deg] with estimated standard deviations: Si–Si (mean) 237.5, Si(2)–C(1) 196.4(2), Si(3)–C(1) 195.1(2), Si–C_ethyl_ (mean) 190.3, C(15)–C(16) 135.0(2), C(13)–C(14) 134.8(2), C(1)–O(1) 144.0(2), O(1)–Si(1) 161.3(1); Si(2)–C(1)–Si(3) 87.7(1), C(1)–Si(3)–C(16) 94.8(1), C(1)–Si(3)–C(22) 98.3(1), C(1)–Si(2)–C(15) 95.1(1), C(1)–Si(2)–C(21) 96.6(1), Si(2)–C(21)–C(22) 112.5(1), C(21)–C(22)–Si(3) 109.4(1), C(22)–Si(3)–C(16) 104.9(1), Si(3)–C(16)–C(15) 112.0(1), C(16)–C(15)–Si(5) 109.9(1), C(15)–Si(2)–C(21) 104.8(1); Si(2)–C(15)–C(16)–Si(3) −2.1(2), Si(2)–C(21)–C(22)–Si(3) −1.5(2), Si(2)–C(15)–C(16)–C(19) 172.8(1), Si(3)–C(16)–C(15)–C(17) −177.5(1), Si(2)–C(21)–C(22)–C(23) 176.1(1), Si(3)–C(22)–C(21)–C(25) −179.6(1).

Crystals of **8a** suitable for X-ray crystallography could be obtained after recrystallization from acetone. The obtained structure is depicted in Figure 4 along with selected structural parameters. The mean C=C bond distance of 134,9 pm compares well with the C=C bond lengths measured for compounds **2**–**5**. The C(1)–Si(2) and C(1)–Si(3) bonds are significantly elongated as compared to the observed endocyclic Si–C(vinyl) bond distances. The Si(2)–C(1)–Si(3) angle of 87.7° is markedly smaller than the ideal tetrahedral angle. In close analogy, the endocyclic Si–C=C angles are reduced by about 14° relative to the structure of (Me_3_Si)_2_C=C(SiMe_3_)_2_.^53^ The environment around the endocyclic C=C bonds is approximately planar with an angle between the Si(2)–C(15)–C(16)–Si(3) and Si(2)–C(21)–C(22)–Si(3) least square planes of 63.8°. These structural features are strongly reminiscent of the crystal structures of 7-silanorbornadiene derivatives^54,55^ and of the gas-phase structure of norbornadiene and indicate strong intramolecular strain.^56^

**Figure 5:**
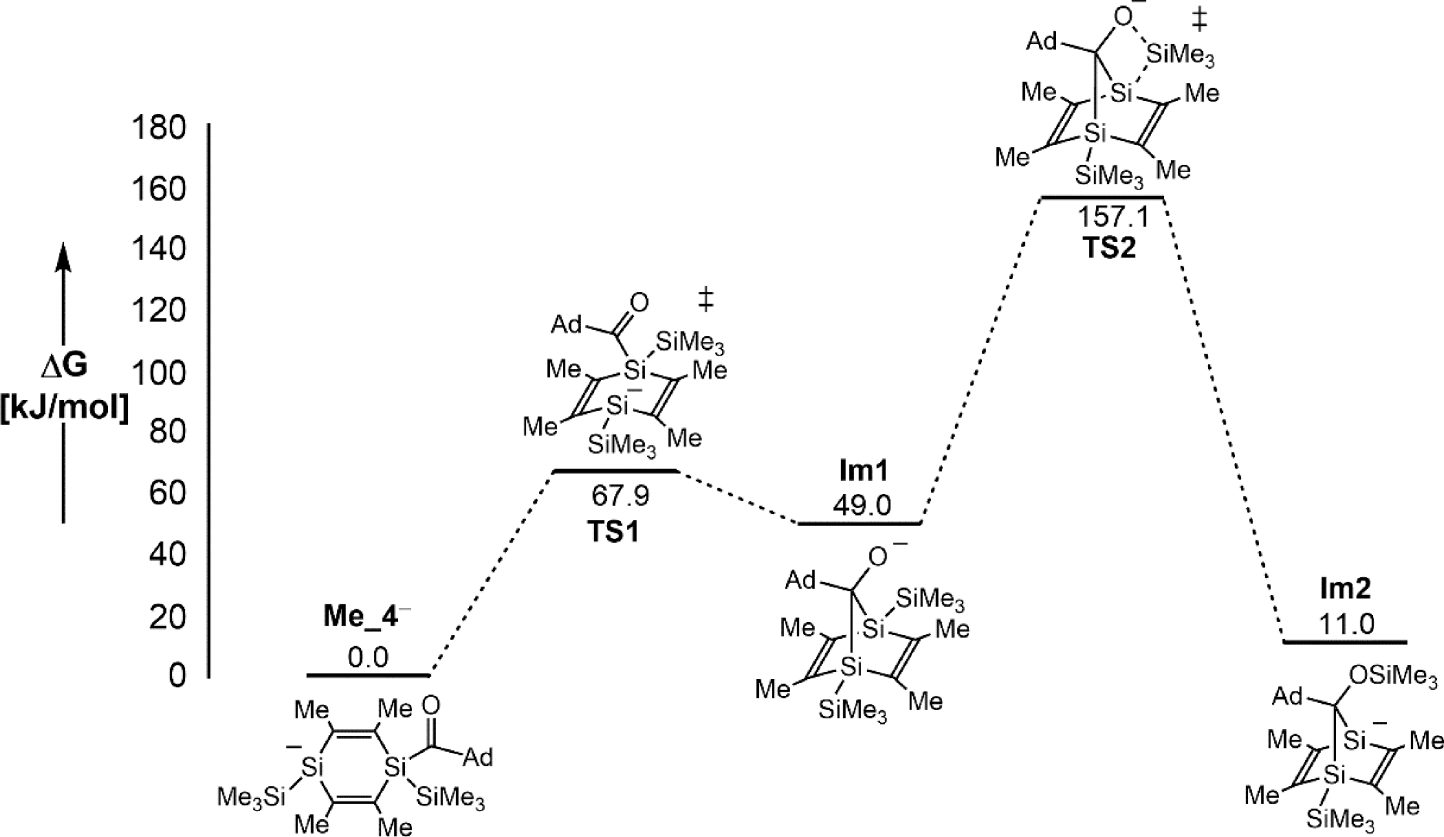
Reaction profile for the disilanorbornadienide formation calculated at the CPCM (THF) B3LYP-GD3/6-31 + G(d) level.

To support the reaction mechanism presented in Scheme 4, the rearrangement cascade responsible for the formation of the disilanorbornadienide skeleton from the acyl-1,4-disilacyclohexadienide anion **4^–^** was calculated at the CPCM (THF) B3LYP-GD3/6-31 + G(d) level of theory. Computations were performed using a simplified model system with the ethyl groups exchanged by methyl and without the potassium counter ion as it is embedded in a 18-cr-6 ligand. The resulting reaction profile is depicted in Figure 5.

The computed reaction cascade starts from the most stable rotamer of **Me_4^–^** with the 1,4-disilacyclohexadienide ring in a slightly distorted chair conformation. In the first reaction step, this rotamer needs to rearrange into the energetically higher boat structure **TS1** to enable the subsequent addition reaction of the negatively charged silicon atom to the carbonyl moiety. This cyclization step features an activation energy of ΔΔ*G*_AE_^0^ = 67.9 kJ mol^–1^ and provides straightforward access to the disilanorbornadienide cage **Im1** with the negative charge localized on the oxygen atom (NPA charge = −0.95). This fact finally leads to the scission of the adjacent Si–Si bond and to the migration of the Me_3_Si group to the oxygen atom under formation of **Im2** with an activation barrier of ΔΔ*G*_AE_^0^ = 108.1 kJ mol^–1^. The significantly higher activation barrier calculated for step two is consistent with our experimental observations. Quenching of the reaction solution with PhMe_2_SiCl at −20 °C affords the norbornadiene **9** with the PhMe_2_Si group in the apical position, while compound **8b** with the PhMe_2_Si group attached to one bridgehead Si atom was obtained after quenching at room temperature (compare Scheme 4).

## Conclusions

To conclude, we have synthesized the first representatives of the previously unknown class of acyl-substituted 1,4-disilacyclohexa-2,5-dienes and investigated their potential as educts for the preparation of 1,4-disilacyclohexadienes with exocyclic Si=C double bonds. Such species are interesting synthetic targets because of unusual electronic properties caused by cyclic crossconjugational-type interactions. Our results demonstrate that compounds of that type are actually formed as the primary products when acyl-1,4-disilylcyclohexa-2,5-dienes were photolyzed with λ > 300 nm light. Such silenes, however, are not stable under the photolytical conditions applied and, thus, only could be characterized by trapping experiments. Attempts to prepare 1,4-disilacyclohexadienes with exocyclic Si=C double bonds by the treatment of acyl-1,4-disilacyclohexa-2,5-dienes with KO^*t*^Bu followed by quenching of the 1,4-disilanorbornadienide intermediates with R_3_SiCl led to the surprising formation of silylated 1,4-disilanorbornadiene cages. In previous studies, structurally related acylcyclohexasilanes cleanly afforded the corresponding methylenecyclohexasilanes under similar conditions. The results of DFT calculations performed to elucidate the mechanism responsible for the unexpected formation of the 1,4-disilanorbornadiene cages are consistent with our experimental results.

## Experimental Section

### General Considerations

All experiments were performed under a nitrogen atmosphere using standard Schlenk or glove-box techniques. Solvents were dried using a column solvent purification system.^57^ Commercial KOtBu (97%) was dissolved in THF and filtered under a nitrogen atmosphere to remove potassium hydroxide and dried by heating to 150 °C in vacuo for 2 h after removal of the solvent prior to use. Otherwise commercially available chemicals were used as purchased. Compound **1** was synthesized according to published procedures.^13^^1^H (299.95 MHz), ^13^C (75.43 MHz), and ^29^Si (59.59 MHz) NMR spectra were recorded on a Varian INOVA 300 spectrometer, using either the internal ^2^H-lock signal of the solvent or a D_2_O capillary as an external lock. Chemical shift values are referenced versus TMS. High-resolution mass spectrometry (HRMS) spectra were recorded on a Kratos Profile mass spectrometer equipped with a solid probe inlet. Melting points were determined in one-side melted off capillaries using a Buechi 535 apparatus and are uncorrected. Elemental analyses were carried out using a Hanau Vario Elementar EL apparatus.

### Synthesis of 1-Potassio-1,4,4-tristrimethylsilyl-1,4-disilacyclohexa-2,5-diene (**1-K**)

**1-K** was prepared by stirring a mixture of 0.50 g (0.97 mmol, 1.00 equiv) of **1**, 0.12 g (1.02 mmol, 1.05 equiv) of KO^*t*^Bu, and 0.28 g (1.07 mmol, 1.07 equiv) of 18-crown-6 dissolved in 10 mL of DME for 1 h at room temperature. After evaporation of the solvent in vacuo, 0.39 g (89%) of the semisolid red-brown product was obtained and characterized by NMR spectroscopy.

^29^Si-NMR (THF-d8, TMS, ppm): −18.5 (SiMe_3_); −58.1 (C=C*Si*C=C). ^13^C-NMR (C_6_D_6_, TMS, ppm): 152.7 (C=C); 27.8 (ethyl-CH_2_); 17.8 (ethyl-CH_3_); 2.6 (SiMe_3_). ^1^H-NMR (C_6_D_6_, TMS, ppm, rel. Int.): 2.45 (q, ^3^*J*_H–H_ = 7.3 Hz, 8 H, ethyl-CH_2_); 1.38 (t, ^3^*J*_H–H_ = 7.3 Hz, 12 H, ethyl-CH_3_); 0.54 (s, 27 H, SiMe_3_).

### Synthesis of 1-Methyl-1,4,4-tristrimethylsilyl-1,4-disilacyclohexa-2,5-diene (**2**)

A DME (10 mL) solution of **1-K** was prepared from 0.50 g (0.97 mmol, 1.0 equiv) of **1**, 0.12 g (1.02 mmol, 1.05 equiv) of KO^*t*^Bu, and 0.28 g (1.07 mmol, 1.10 equiv) of 18-crown-6 as described above and slowly added to a DME solution of 0.5 mL of Me_2_SO_4_ (in excess) at 0 °C. After warming to room temperature, aqueous work up was accomplished with 10% sulfuric acid. Phase separation, drying of the organic layer over Na_2_SO_4_, and removal of the solvent in vacuo followed by recrystallization of the resulting solid product from acetone at −30 ° C afforded 0.29 g (66%) of pure **2** as colorless crystals.

Mp: 135–136 °C. Anal. calcd. For C_22_H_50_Si_5_: C, 58.07; H, 11.08. found: C, 58.10; H, 11.08. ^29^Si{^1^H}-NMR (C_6_D_6_, TMS, ppm): −17.3, −17.5, −20.3 (SiMe_3_); −31.6 (*Si*MeSiMe_3_); −52.2 (*Si*(SiMe_3_)_2_). ^13^C{^1^H}-NMR (C_6_D_6_, TMS, ppm): 152.0, 151.4 (C=C); 26.3, 25.2 (ethyl-CH_2_); 15.6, 15.2 (ethyl-CH_3_); 2.0, 0.2, −0.9 (SiMe_3_); −5.2 (SiMe). ^1^H-NMR (C_6_D_6_, TMS, ppm, rel. Int.): 2.63–2.44 (m, 4H, ethyl-CH_2_); 1,88–1.75 (m, 8H, ethyl-CH_2_); 1.05 (t, ^3^*J*_H–H_ = 7.4 Hz, 6H, ethyl-CH_3_); 1.01 (t, ^3^*J*_H–H_ = 7.5 Hz, 6H, ethyl-CH_3_); 0.40 (s, 3H, SiMe); 0.39, 0.22, 0.15 (s, 9H each, SiMe_3_). MS: calc. For [C_22_H_50_Si_5_]^·^^+^ (M^+^): 454.2759, found: [*m/e* (relative intensity)] 454.3 (21.4%, (M^+^)).

### Synthesis of 1-Phenyldimethylsilyl-1,4,4-tristrimethylsilyl-1,4-disilacyclohexa-2,5-diene (**3**)

**3** was prepared according to the synthetic protocol for **2** with 0.50 g (0.97 mmol, 1.00 equiv) of **1**, 0.12 g of KO^*t*^Bu (1.02 mmol, 1.05 equiv), 0.28 g (1.07 mmol, 1.10 equiv) of 18-crown-6, and 1 mL of PhMe_2_SiCl (in excess). After recrystallization of the crude product from acetone at −30 ° C, 0.20 g (36%) of pure **3** was obtained as colorless crystals.

Mp: 112–113 °C. Anal. calcd. For C_29_H_58_Si_6_: C, 60.55; H, 10.16. found: C, 60.45; H, 10.12. ^29^Si{^1^H}-NMR (C_6_D_6_, TMS, ppm): −17.7, −17.8, −18.1 (SiMe_3_); −53.1, −53.3 (*Si*(SiMe_3_)_2_, *Si*(SiMe_3_SiMe_2_Ph)); −21.0 (*Si*Me_2_Ph). ^13^C{^1^H}-NMR (C_6_D_6_, TMS, ppm): 152.0, 151.4 (C=C); 141.0, 134.6, 128.8, 127.9(Ph); 26.5, 26.4 (ethyl-CH_2_); 15.4 (ethyl-CH_3_); 1.9, 1.6, 0.9 (SiMe_3_); −0.8 (SiMe). ^1^H-NMR (C_6_D_6_, TMS, ppm, rel. Int.): 7.61–7.55 (m, 2H, aryl-H); 7.27–7.18 (m, 3H, aryl-H); 2.40–2.20 (m, 4H ethyl-CH_2_); 2.03–1.92 (m, 2H ethyl-CH_2_); 1.89–1.78 (m, 2H ethyl-CH_2_); 1.07 (t, ^3^*J*_H–H_ = 7.4 Hz, 6H, ethyl-CH_3_); 0.97 (t, ^3^*J*_H–H_ = 7.4 Hz, 6H, ethyl-CH_3_); 0.52 (s, 6H, SiMe_2_Ph); 0.39, 0.31, 0.26 (s, 9H each, SiMe_3_). MS: calc. For [C_29_H_58_Si_6_]^·^^+^ (M^+^): 574.3154, found: [*m/e* (relative intensity)] 574.4 (32.4%, (M^+^).

### Synthesis of 1-Adamantoyl-1,4,4-tristrimethylsilyl-1,4-disilacyclohexa-2,5-diene (**4**)

A DME solution (25 mL) of **1-K** prepared as described above from 0.30 g (0.58 mmol, 1.00 equiv) of **1**, 0.07 g (0.60 mmol, 1.05 equiv) of KO^*t*^Bu, and 0.16 g (0.65 mmol, 1.10 equiv) of 18-crown-6 was slowly added to a solution of 0.13 g (0.65 mmol, 1.10 equiv) of ClCOAd in 20 mL of DME at −50 ° C. After warming to room temperature, aqueous work up was accomplished with 10% H_2_SO_4_. Drying of the organic layer over Na_2_SO_4_ and removal of the solvent in vacuo followed by recrystallization of the resulting solid product from acetone at −30 °C afforded 0.25 g (71%) of yellow crystals of pure **4**.

Mp: 171–173 °C. Anal. calcd. For C_32_H_62_OSi_5_: C, 63.71; H, 10.36. found: C, 63.46; H, 9.74. ^29^Si{^1^H}-NMR (C_6_D_6_, TMS, ppm): −16.5, −16.8, −17.2, (SiMe_3_); −36.4 (*Si*(SiMe_3_CO)); −52.7 (*Si*(SiMe_3_)_2_). ^13^C{^1^H}-NMR (C_6_D_6_, TMS, ppm): 247.9 (C=O); 154.5, 151.3 (C=C); 54.1, 38.1, 37.1, 28.7 (Ad-C); 26.6, 26.3 (ethyl-CH_2_); 15.6, 14.7 (ethyl-CH_3_); 2.1, 1.3, 0.9 (SiMe_3_). ^1^H-NMR (C_6_D_6_, TMS, ppm, rel. Int.): 2.50–2.30 (broad, 6H, ethyl-CH_2_); 2.25–2.10 (broad, 6H, ethyl-CH_2_); 2.05–1.85 (broad, 11H, Ad-CH + Ad-CH_2_ + ethyl-CH_2_); 1.75–1.55 (broad, 6H, Ad-CH_2_); 1.15–0.95 (broad, 12 H, ethyl-CH_3_); 0.48, 0.37, 0.22 (s, 9H each, SiMe_3_). HRMS: calc. For [C_32_H_62_OSi_5_]^·^^+^ (M^+^): 602.3647; found, 602.3651.

### Synthesis of 1-Mesitoyl-1,4,4-tristrimethylsilyl-1,4-disilacyclohexa-2,5-diene (**5**)

**5** was prepared according to the synthetic protocol for **4** with 2.00 g (3.89 mmol, 1.00 equiv) of **1**, 0.46 g (4.09 mmol, 1.05 equiv) of KO^*t*^Bu, 1.13 g (4.29 mmol, 1.10 equiv) of 18-crown-6, and 0.78 g (4.29 mmol, 1.10 equiv) of ClCOMes. Recrystallization of the crude product from acetone at −30 ° C afforded 1.32 g (56%) of yellow crystals of pure **5**.

Mp: 161–163 °C. Anal. calcd. For C_31_H_58_OSi_5_: C, 63.41; H, 9.96. Found: C, 63.01; H, 9.73. ^29^Si{^1^H}-NMR (C_6_D_6_, TMS, ppm): −16.0, −16.4, −17.1, (SiMe_3_); −42.0 (*Si*(SiMe_3_CO)); −51.9 (*Si*(SiMe_3_)_2_). ^13^C{^1^H}-NMR (C_6_D_6_, TMS, ppm): 248.8 (C=O); 155.2, 151.2 (C=C); 145.3, 138.3, 133.8, 129.2 (aryl-C); 27.1, 25.9 (ethyl-CH_2_); 21.1 (aryl-CH_3_); 14.9, 14.7 (ethyl-CH_3_); 1.9, 1.5, 0.7 (SiMe_3_). ^1^H-NMR (C_6_D_6_, TMS, ppm, rel. Int.): 6.58 (s, 2H, aryl-H); 2.40–2.25 (broad, 10H, ethyl-CH_2_ + aryl-CH_3_); 2.20–2.05 (m, 4H, ethyl-CH_2_); 2.03 (s, 3H, aryl-CH_3_); 0.92 (t, ^3^*J*_H–H_ = 7.3 Hz, 6H, ethyl-CH_3_); 0.90 (t, ^3^*J*_H–H_ = 7.3 Hz, 6H, ethyl-CH_3_); 0.41, 0.30, 0.27 (s, 9H each, SiMe_3_). HRMS: calc. For [C_31_H_58_OSi_5_]^·^^+^ (M^+^), 586.3334; found, 586.3212.

### Photolysis of **4** in Benzene

First, 0.10 g of **4** was dissolved in 0.6 mL of C_6_D_6_ and irradiated in a Pyrex glass NMR tube with a 150 W high-pressure Hg lamp at room temperature. NMR analysis performed after 30 and 90 min showed the formation of increasing amounts of undefined material. Low-field shifted ^13^C resonance lines typical for silenes could not be detected.

### Photolysis of **4** in the Presence of MeOH/Et_3_N

A solution of 0.10 g (0.17 mmol) of **4** and 1 drop of anhydrous Et_3_N in 0.6 mL of C_6_D_6_ and 1 drop of methanol was placed in a Pyrex glass NMR tube and photolyzed at 25 °C with a 150 W mercury lamp for 30 min. NMR analysis performed after removal of the volatile components in vacuo showed the predominant formation of **6** along with smaller amounts of unidentified byproducts. Small amounts of pure **6** could be isolated in low yield (<10%) as a colorless oil from the crude photolysis solution by preparative thin-layer chromatography over silica gel with heptane as the eluant and characterized by NMR spectroscopy and GC–MS analysis.

^29^Si{^1^H}-NMR (C_6_D_6_, TMS, ppm): 15.1 (OSiMe_3_); −15.9, −16.6 (Si*Si*Me_3_); −20.1 (*Si*OMe); −55.9 (*Si*(SiMe_3_)_2_). ^13^C{^1^H}-NMR (C_6_D_6_, TMS, ppm): 156.6, 156.4, 154.1, 154.0 (C=C); 77.1 (*C*H(*Ad*)OSi), 50.7 (O*C*H_3_); 40.5, 37.7, 29.2 (Ad); 26.9, 26.6, 25.2, 25.0 (ethyl-CH_2_); 15.2, 15.1, 14.6, 14.5 (ethyl-CH_3_); 2.3, 1.2, 1.1 (SiMe_3_). ^1^H-NMR (C_6_D_6_, TMS, ppm, rel. Int.): 3.57 (1H, s, C*H*(*Ad*)OSi); 3.37 (3H, s, OC*H*_3_); 2.93–2.81 (m, 1H, ethyl-CH_2_); 2.48–2.38 (2 × m, 5H, ethyl-CH_2_); 2.30–2.18 (m, 2H, ethyl-CH_2_); 2.05–1.95 (broad, 3H, Ad-C*H*); 1.80–1.65 (broad, 12H, Ad-C*H*_2_); 1.21 (t, ^3^*J*_H–H_ = 7.5 Hz, 6H, ethyl-CH_3_); 1.06 (t, ^3^*J*_H–H_ = 7.4 Hz, 6H, ethyl-CH_3_); 1.00 (t, ^3^*J*_H–H_ = 7.4 Hz, 6H, ethyl-CH_3_); 0.36 (s, 18H, SiMe_3_), 0.22 (s, 9H, SiMe_3_). MS: calc. For [C_32_H_634_O_2_Si_5_]^·^^+^ (M^+^ – CH_3_): 619.37, found: [*m/e* (relative intensity)] 619.5 (0.6%).

### Photolysis of **5**

First, 0.20 g (0.34 mmol) of **5** was dissolved in 0.6 mL of C_6_D_6_ and irradiated in a NMR tube with a 150 W high-pressure Hg lamp for 60 min at room temperature. NMR analysis performed after this time showed the formation of a new product. After removal of the solvent in vacuo, 0.13 g (65%) of pure **7** was isolated as a colorless oil by preparative thin-layer chromatography over silica gel with heptane as the eluent.

Anal. calcd. For C_31_H_58_OSi_5_: C, 63.41; H, 9.96. Found: C, 63.20; H, 9.81. ^29^Si{^1^H}-NMR (C_6_D_6_, TMS, ppm): 13.1 (OSiMe_3_); −15.9, −16.8 (Si*Si*Me_3_); −43.5 (*Si*HCO); −51.7 (*Si*(SiMe_3_)_2_). ^13^C{^1^H}-NMR (C_6_D_6_, TMS, ppm): 154.4, 153.4, 151.8, 150.1 (C=C); 146.8, 139.7, 137.9, 132.3, 129.0, 121.1 (aryl-C); 75.4 (Si*C*O); 41.5 (aryl-CH_2_); 26.1, 25.9, 25.8, 24.3 (ethyl-CH_2_); 21.8, 16.9 (aryl-CH_3_); 15.3, 14.7, 14.5, 14.4 (ethyl-CH_3_); 1.7, 1.6, 0.0 (SiMe_3_). ^1^H-NMR (C_6_D_6_, TMS, ppm, rel. Int.): 6.72, 6.64 (s, 1H each, aryl-H); 4.99 (s, 1 H, Si-H); 3.70 (d, ^2^*J*_H–H_ = 14.1 Hz, 1H, endocyclic CH_2_); 3.13 (d, ^2^*J*_H–H_ = 14.1 Hz, 1H, endocyclic CH_2_); 2.90–2.51 (3 × m, 3H, ethyl-CH_2_); 2.39, 2.16 (s, 3H each, aryl-CH_3_); 2.38–2.26 (m, 2H, ethyl-CH_2_); 1.94–1.83 (m, 1H, ethyl-CH_2_); 1.79–1.68 (m, 1H, ethyl-CH_2_); 1.23 (t, ^3^*J*_H–H_ = 7.5 Hz, 3H, ethyl-CH_3_); 1.11 (t, ^3^*J*_H–H_ = 7.4 Hz, 3H, ethyl-CH_3_); 0.94 (t, ^3^*J*_H–H_ = 7.5 Hz, 3H, ethyl-CH_3_); 0.84 (t, ^3^*J*_H–H_ = 7.4 Hz, 3H, ethyl-CH_3_); 0.43, 0.18, 0.09 (s, 9H each, SiMe_3_). MS: calc. For [C_31_H_58_OSi_5_]^·^^+^ (M^+^): 586.3334; found: [*m/e* (relative intensity)] 586.3 (3.7%).

### Reaction of **4** with KO^*t*^Bu/Me_3_SiCl

A DME (10 mL) solution of 0.60 g (0.10 mmol, 1.00 equiv) of **4**, 0.12 g (0.11 mmol, 1.05 equiv) of KO^*t*^Bu, and 0.28 g (0.11 mmol, 1.10 equiv) of 18-crown-6 was stirred for 1 h at 0 °C and for another 2 h at room temperature. After this time, the resulting red mixture was added slowly to a solution of 0.12 g (0.65 mmol, 1.10 equiv) of Me_3_SiCl in 10 mL of pentane at 0 °C. After warming to room temperature, aqueous work up was accomplished with 10% H_2_SO_4_. Phase separation, drying of the organic layer over Na_2_SO_4_, and removal of the solvent in vacuo followed by recrystallization of the resulting solid product from acetone at −30 ° C afforded 0.37 g (62%) of colorless crystals of pure **8a**.

Mp: 224–227 °C. Anal. calcd. For C_32_H_62_OSi_5_: C, 63.71; H, 10.36. Found: C, 63.62; H, 10.04. ^29^Si{^1^H}-NMR (C_6_D_6_, TMS, ppm): 0.7 (OSiMe_3_); −20.3, (Si*Si*Me_3_); −24.7 (*Si*SiMe_3_). ^13^C{^1^H}-NMR (C_6_D_6_, TMS, ppm): 161.3, 160.5 (C=C); 113.7 (*C*OSiMe_3_); 44.6, 42.1, 37.5, 29.8 (Ad-C); 26.4, 24.9 (ethyl-CH_2_); 16.1, 15.9 (ethyl-CH_3_); 3.7, 2.1 (SiMe_3_). ^1^H-NMR (C_6_D_6_, TMS, ppm, rel. Int.): 2.75–2.54 (2 × m, 4H, ethyl-CH_2_); 2.30–2.16 (m, 4H, ethyl-CH_2_); 2.09–2.02, (broad, 3H, Ad-H); 1.87–1.81 (broad, 6H, Ad-H); 1.79–1.65 (b, 6H, Ad-H); 1.05–0.99 (m, 12H, ethyl-CH_3_); 0.47 (s, 18H, SiSiMe_3_); 0.21 (s, 9H, OSiMe_3_). MS: calc. For [C_32_H_62_OSi_5_]^·^^+^ (M^+^): 602.3647, found: [m/e (relative intensity)] 602.4 (0.3%), (M^+^); 587.4 (3.0%), M^+^ – CH_3_).

### Reaction of **4** with KO^*t*^Bu/PhMe_2_SiCl

**8b** was prepared according to the synthetic protocol for **8a** with 0.60 g (0.10 mmol, 1.00 equiv) of **4**, 0.12 g (0.11 mmol, 1.05 equiv) of KO^*t*^Bu, 0.28 g (0.11 mmol, 1.10 equiv) of 18-crown-6, and 0.13 g (0.76 mmol, 1.10 equiv) of Me_2_PhSiCl. Recrystallization of the crude product from acetone at −30 ° C afforded 0.47 g (71%) of colorless crystals of pure **8b**.

Mp: 202–203 °C. Anal. calcd. For C_37_H_64_OSi_5_: C, 66.79; H, 9.70. Found: C, 66.44; H, 9.52. ^29^Si{^1^H}-NMR (C_6_D_6_, TMS, ppm): 1.1 (OSiMe_3_); −20.1 (Si*Si*Me_3_); −23.3 (SiMe_2_Ph); −25.0, −25.3 (*Si*SiR_3_). ^13^C{^1^H}-NMR (C_6_D_6_, TMS, ppm): 162.1, 161.2, 160.5, 159.7 (C=C); 139.8, 135.1, 129.1, 128.2 (aryl-C); 114.3 (*C*OSiMe_3_); 44.5, 42.1, 37.5, 29.8 (Ad-C); 26.6, 26.3, 25.1, 25.0 (ethyl-CH_2_); 16.1, 16.05, 15.9, 15.8 (ethyl-CH_3_); 3.6, 2.3, (SiMe_3_); 0.7, 0.6 (SiMe_2_Ph). ^1^H-NMR (C_6_D_6_, TMS, ppm, rel. Int.): 7.75–7.65 (m, 2 H, aryl-H); 7.27–7.18 (m, 3 H, aryl-H); 2.73–2.55 (m, 4 H ethyl-CH_2_); 2.41–2.18 2 × (m, 4H, ethyl-CH_2_); 2.08–2.00, (broad, 3H, Ad-H); 1.85–1.80 (broad, 6H, Ad-H); 1.71–1.65 (broad, 6H, Ad-H); 1.04–0.96 (m, 9H, ethyl-CH_3_); 0.80–0.70 (broad, 9H, SiMe_2_Ph + ethyl-CH_3_); 0.46 (s, 9H, SiSiMe_3_); 0.15 (s, 9H, OSiMe_3_). MS: calc. For [C_37_H_64_OSi_5_]^·^^+^ (M^+^ – CH_3_): 649,3569, found: [*m/e* (relative intensity)] 649.5 (4.8%).

### Reaction of **4** with KO^*t*^Bu/PhMe_2_SiCl at Low Temperatures

A DME (10 mL) solution of 0.60 g (0.10 mmol, 1.00 equiv) of **4**, 0.12 g (0.11 mmol, 1.05 equiv) of KO^*t*^Bu, and 0.28 g (0.11 mmol, 1.10 equiv) of 18-crown-6 was stirred for 15 min at −20 ° C. After this time, the resulting yellow mixture was added to a solution of 0.13 g (0.76 mmol, 1.20 equiv) of Me_2_PhSiCl in 10 mL of pentane at 0 °C. After warming to room temperature, aqueous work up was accomplished with 10% H_2_SO_4_. Phase separation, drying of the organic layer over Na_2_SO_4_, and removal of the solvent in vacuo followed by recrystallization of the resulting solid product from acetone at −30 °C afforded 0.31 g (47%) of colorless crystals of pure **9**.

Mp: 202–203 °C. Anal. calcd. For C_37_H_64_OSi_5_: C, 66.79; H, 9.70. Found: C, 66.20; H, 9.78. ^29^Si{^1^H}-NMR (C_6_D_6_, TMS, ppm): −8.4 (OSiMe_2_Ph); −20.3 (Si*Si*Me_3_); −24.5 (*Si*SiMe_3_). ^13^C{^1^H}-NMR (C_6_D_6_, TMS, ppm): 161.4, 160.6 (C=C); 141.4, 134.1, 129.2, 127.9 (aryl-C); 114.6 (*C*OSiMe_3_); 44.3, 42.1, 37.3, 29.7 (Ad-C); 26.4, 24.9 (ethyl-CH_2_); 16.2, 15.9 (ethyl-CH_3_); 2.1, 1.7 (SiMe_3,_ SiMe_2_Ph. ^1^H-NMR (C_6_D_6_, TMS, ppm, rel. Int.): 7.75–7.68 (m, 2H, aryl-H); 7.32–7.20 (m, 3H, aryl-H); 2.78–2.68 (m, 2H ethyl-CH_2_); 2.64–2.53 (m, 2 H ethyl-CH_2_); 2.30–2.16 (m, 4H, ethyl-CH_2_); 2.00–1.90, (broad, 3H, Ad-H); 1.81–1.71 (broad, 6H, Ad-H); 1.66–1.55 (broad, 6H, Ad-H); 1.02 (t, (t, broad, ^3^*J*_H–H_ = 7.3 Hz, 12H, ethyl-CH_3_); 0.46, 0.45 (s, 24H, SiMe_2_Ph + SiMe_3_). MS: calc. For [C_37_H_64_OSi_5_]^·^^+^ (M^+^ – CH_3_): 649,3569, found: [*m/e* (relative intensity)] 649.5 (2.0%).

### Computational Studies

All calculations were performed with the Gaussian16 program suite.^58^ Geometries were optimized using the B3LYP functional with empirical dispersion corrections D3,^59^ denoted as B3LYP-GD3 in the text, together with 6-31 + G(d) basis sets. Solvent effects were considered for geometry optimizations and vibrational frequency calculations using the self-consistent reaction field method based on the CPCM method for THF.^60,61^ The nature of stationary points was verified by vibrational frequency calculations. The magnetic shieldings were calculated with the mPW1PW91 hybrid functional in combination with IGLO-II basis sets.^62^ The reference molecule tetramethylsilane has a ^29^Si magnetic shielding of 349.5 ppm at this level of theory.

### X-ray Crystallography

All crystals suitable for single-crystal X-ray diffractometry were removed from a vial or a Schlenk tube and immediately covered with a layer of silicone oil. A single crystal was selected, mounted on a glass rod on a copper pin, and placed in the cold N_2_ stream provided by an Oxford Cryosystems cryostream. XRD data collection was performed using a Bruker APEX II diffractometer with use of an IμS microsource (Incoatec microfocus) sealed tube of Mo Kα radiation (λ = 0.71073 Å) and a CCD area detector. Data integration was carried out using SAINT.^63^ Empirical absorption corrections were applied using SADABS.^64,65^ The structures were solved with use of the intrinsic phasing option in SHELXT^66^ and refined by the full-matrix least-squares procedures in SHELXL^67−70^ as implemented in the program SHELXLE.^71^ The space group assignments and structural solutions were evaluated using PLATON.^72,73^ Nonhydrogen atoms were refined anisotropically. Hydrogen atoms were located in calculated positions corresponding to standard bond lengths and angles and refined using a riding model. Disorder was handled by modeling the occupancies of the individual orientations using free variables to refine the respective occupancy of the affected fragments (PART).^74^ In some cases, the similarity SAME restraint, the similar-ADP restraint SIMU, and the rigid-bond restraint DELU, as well as the constraints EXYZ and EADP, were used in modeling disorder to make the ADP values of the disordered atoms more reasonable. Disordered positions in the adamantyl moiety of compound **4** were refined using 50/50 split positions. CIF files were edited, validated, and formatted either with the programs encifer,^75^ publCIF,^76^ or Olex2.^77^

## Supporting Information Available

Experimental NMR spectra, crystal structures of **2** and **3**, calculated structures of **1-K**, **4**, and **5**, and calculated NPA charges of **Me_4^–^**, **Im1,** and **Im2** (PDF)
Cartesian coordinates of calculated structures (XYZ)

## Supplementary Material

om2c00475_si_001.pdf

om2c00475_si_002.xyz
