# Supplementary material for: Synthesis and Unusual Reactivity of Acyl-Substituted 1,4-Disilacyclohexa-2,5-dienes
Source: Organometallics. 2022 Nov 14;41(23):3686–96. doi: 10.1021/acs.organomet.2c00475 (PMC9749028; doi:10.1021/acs.organomet.2c00475)
Supplement: Supplementary file 1 — om2c00475_si_001.pdf [file om2c00475_si_001.pdf]

Supporting Information

Synthesis and Unusual Reactivity of Acyl-Substituted 1,4-disilacyclohexa-2,5-dienes

Lukas Schuh, Ana Torvisco, Michaela Flock, Christa Grogger
and Harald Stueger*

Institute of Inorganic Chemistry, Graz University of Technology, Stremayrgasse 9, 8010 Graz, Austria

corresponding author email adress: harald.stueger@tugraz.at

Table of Contents

Inhalt

Figure S1. ^{1}H-NMR spectrum of **1-K** (300 MHz, C_6D_6 solution) S4

Figure S2. ^{13}C{^{1}H}-NMR spectrum of **1-K** (75 MHz, THF-d8 solution) S4

Figure S3. ^{29}Si{^{1}H}-INEPT-NMR spectrum of **1-K** (60 MHz, THF-d8 solution) S5

Figure S4. ^{1}H-NMR spectrum of **2** (300 MHz, C_6D_6 solution) S5

Figure S5. ^{13}C{^{1}H}-NMR spectrum of **2** (75 MHz, C_6D_6 solution) S6

Figure S6. ^{29}Si{^{1}H}-DEPT-NMR spectrum of **2** (60 MHz, C_6D_6 solution) S6

Figure S7. ^{1}H-NMR spectrum of **3** (300 MHz, C_6D_6 solution) S7

Figure S8. ^{13}C{^{1}H}-NMR spectrum of **3** (75 MHz, C_6D_6 solution) S7

Figure S9. ^{29}Si{^{1}H}-DEPT-NMR spectrum of **3** (60 MHz, C_6D_6 solution) S8

Figure S10. ^{1}H-NMR spectrum of **4** (300 MHz, C_6D_6 solution) S8

Figure S11. ^{13}C{^{1}H}-NMR spectrum of **4** (75 MHz, C_6D_6 solution) S9

Figure S12. ^{29}Si{^{1}H}-INEPT-NMR spectrum of **4** (60 MHz, C_6D_6 solution) S9

Figure S13. ^{1}H-NMR spectrum of **5** (300 MHz, C_6D_6 solution) S10

Figure S14. ^{13}C{^{1}H}-NMR spectrum of **5** (75 MHz, C_6D_6 solution) S10

Figure S15. ^{29}Si{^{1}H}-INEPT-NMR spectrum of **5** (60 MHz, C_6D_6 solution) S11

Figure S16. ^{1}H-NMR spectrum of **4** after irradiation with λ >300 nm light S11

Figure S17. ^{13}C-NMR spectrum of **4** after irradiation with λ >300 nm light S12

Figure S18. ^{1}H-NMR spectrum of **6** (300 MHz, C_6D_6 solution) S12

Figure S19. ^{13}C{^{1}H}-NMR spectrum of **6** (75 MHz, C_6D_6 solution) S13

Figure S20. ^{29}Si{^{1}H}-INEPT-NMR spectrum of **6** (60 MHz, C_6D_6 solution) S13

Figure S21. ^{1}H-NMR spectrum of **7** (300 MHz, C_6D_6 solution) S13

Figure S22. ^{13}C{^{1}H}-NMR spectrum of **7** (75 MHz, C_6D_6 solution) S14

Figure S23. ^{29}Si{^{1}H}-and ^{29}Si{^{1}H} DEPT-NMR spectra of **7** (60 MHz, C_6D_6 solution) S15

Figure S24. ^{1}H-NMR spectrum of **8a** (300 MHz, C_6D_6 solution) S15

Figure S25. ^{13}C{^{1}H}-NMR spectrum of **8a** (75 MHz, C_6D_6 solution) S16

Figure S26. ^{29}Si{^{1}H}-INEPT-NMR spectrum of **8a** (60 MHz, C_6D_6 solution) S16

Figure S27. ^{1}H-NMR spectrum of **8b** (300 MHz, DME solution) S17

Figure S28 ^{13}C{^{1}H}-NMR spectrum of **8b** (75 MHz, DME solution) S17

Figure S29. ^{29}Si{^{1}H}-INEPT-NMR spectrum of **8b** (60 MHz, C_6D_6 solution) S18

Figure S30. ^{1}H-NMR spectrum of **9** (300 MHz, C_6D_6 solution) S18

Figure S31. ^{13}C{^{1}H}-NMR spectrum of **9** (75 MHz, C_6D_6 solution) S19

Figure S32. ^{29}Si{^{1}H}-INEPT-NMR spectrum of **9** (60 MHz, C_6D_6 solution) S19

Figure S33. Molecular structure of **2** S20

Figure S34. Molecular structure of **3** S21

Figure S35. Most stable calculated conformers and relative energies of **1-K**........................ S22
Figure S36. Calculated structure of the most stable conformer of **4**................................... S22
Figure S37. Calculated structure of the most stable conformer of **5**................................... S23
Figure S38. Calculated NPA charges of **Me_4**$^-$, **Im1** and **Im2**. S24
Figure S39. UV absorption spectra of compounds **4** and **5**……………….………….……….…….……….…….……S25

Figure S1. ^{1}H-NMR spectrum of **1-K** (300 MHz, C_6D_6 solution)

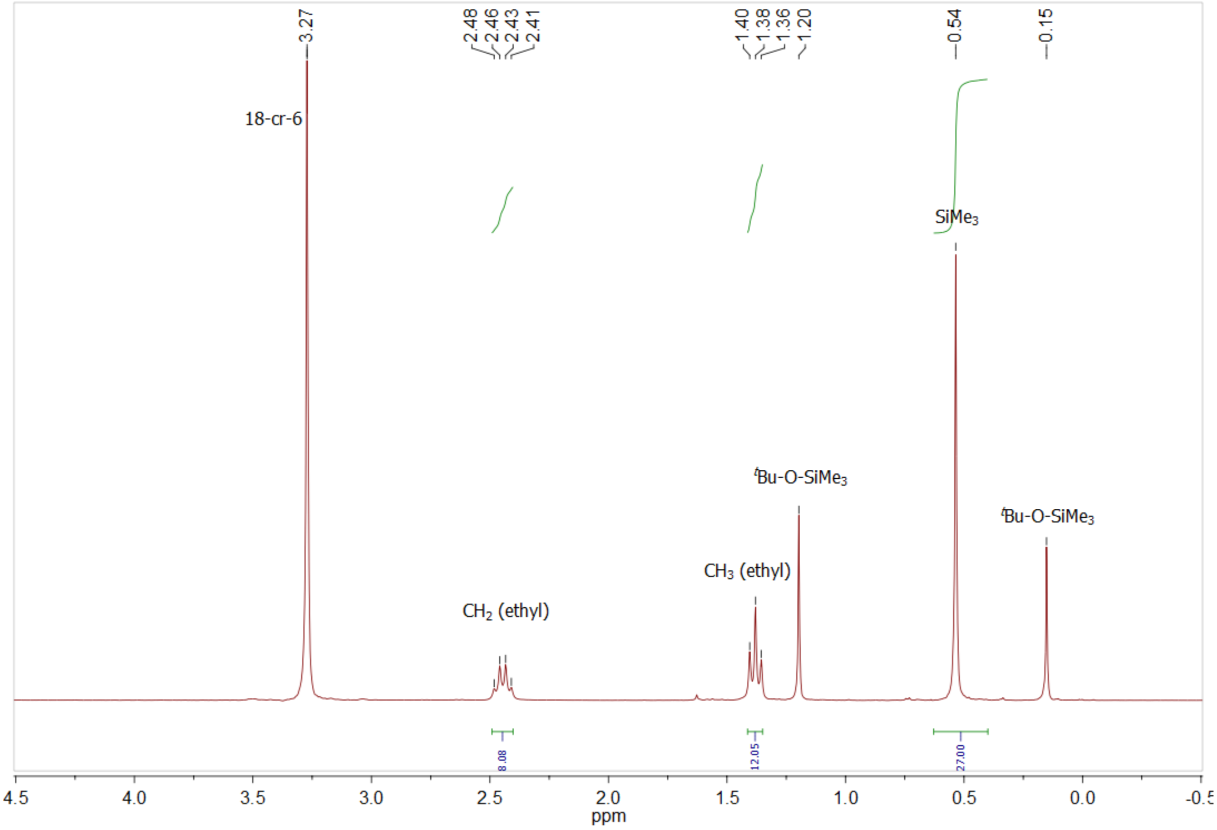

Figure S2. ^{13}C{^{1}H}-NMR spectrum of **1-K** (75 MHz, THF-d8 solution)

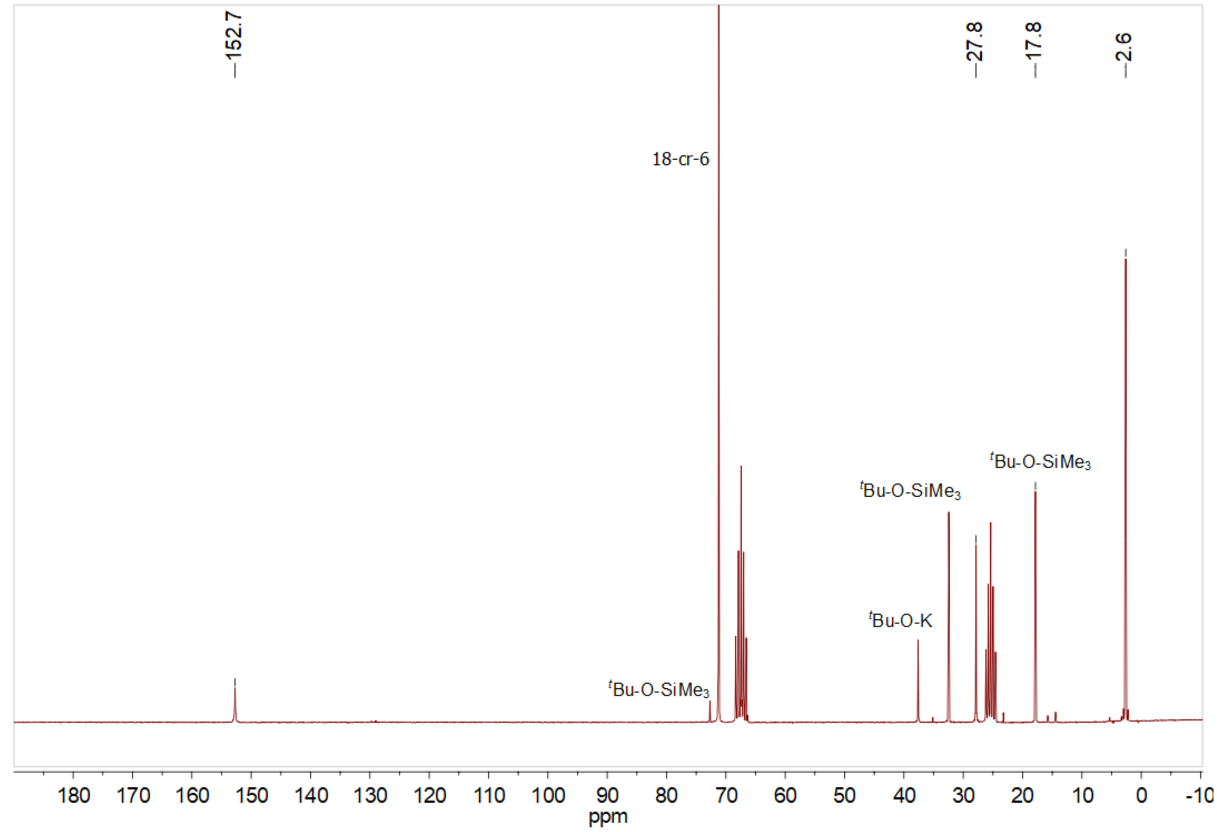

Figure S3. $^{29}Si\{^{1}H\}$-INEPT-NMR spectrum of **1-K** (60 MHz, THF-d8 solution)

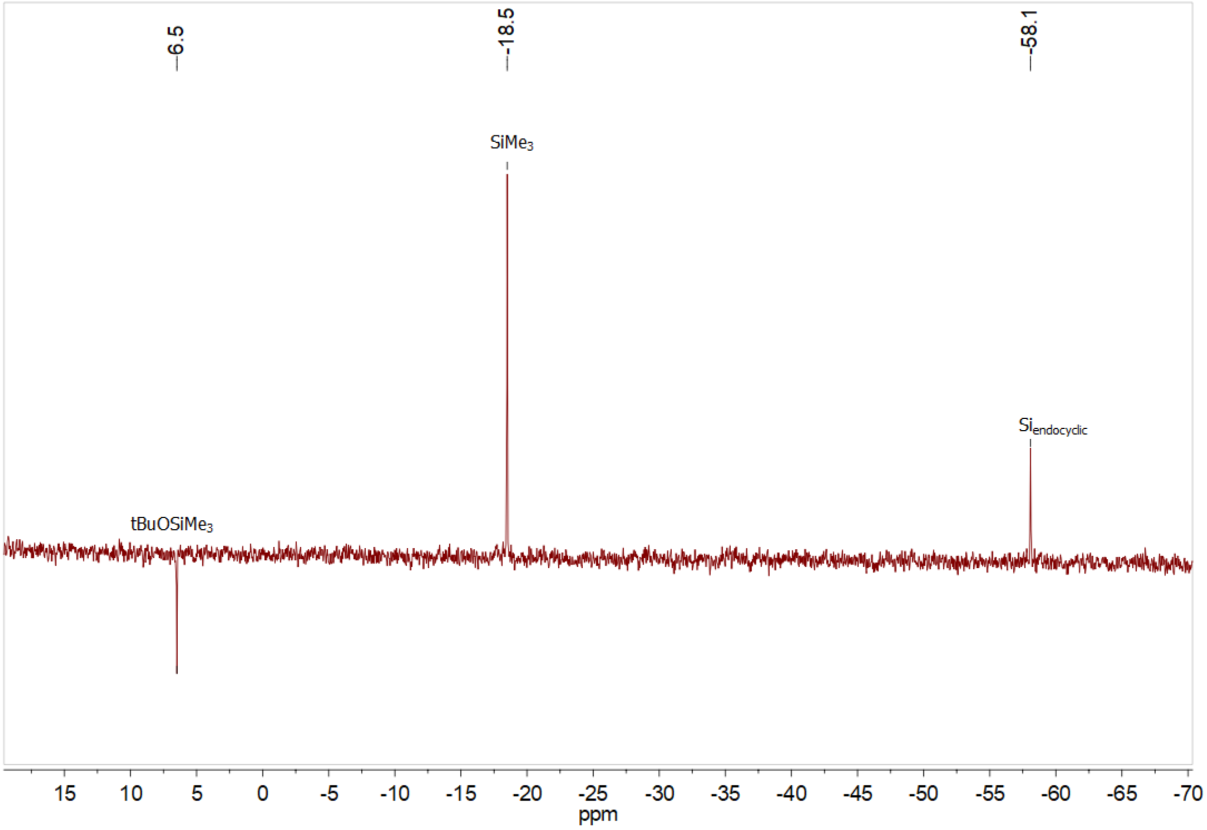

Figure S4. ^{1}H-NMR spectrum of **2** (300 MHz, C_6D_6 solution)

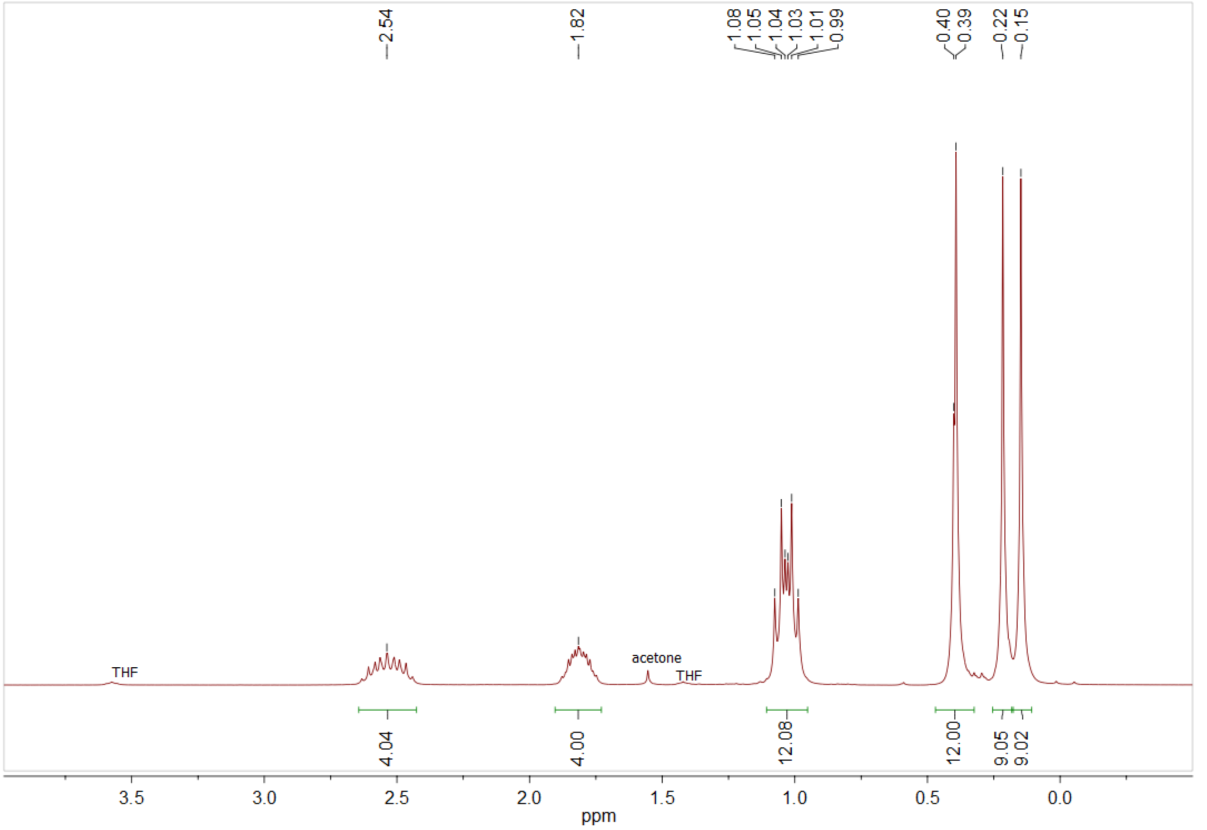

Figure S5. $^{13}C\{^{1}H\}$-NMR spectrum of **2** (75 MHz, C_6D_6 solution)

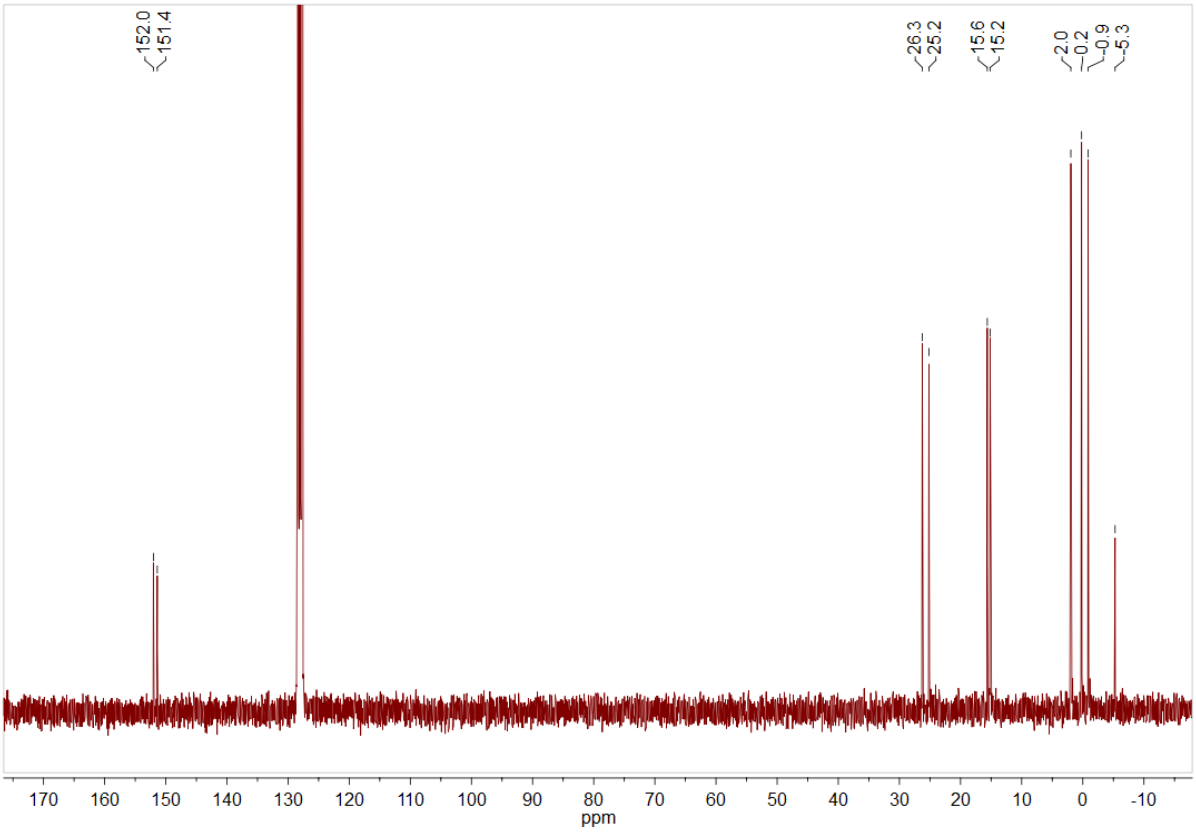

Figure S6. $^{29}Si\{^{1}H\}$-DEPT-NMR spectrum of **2** (60 MHz, C_6D_6 solution)

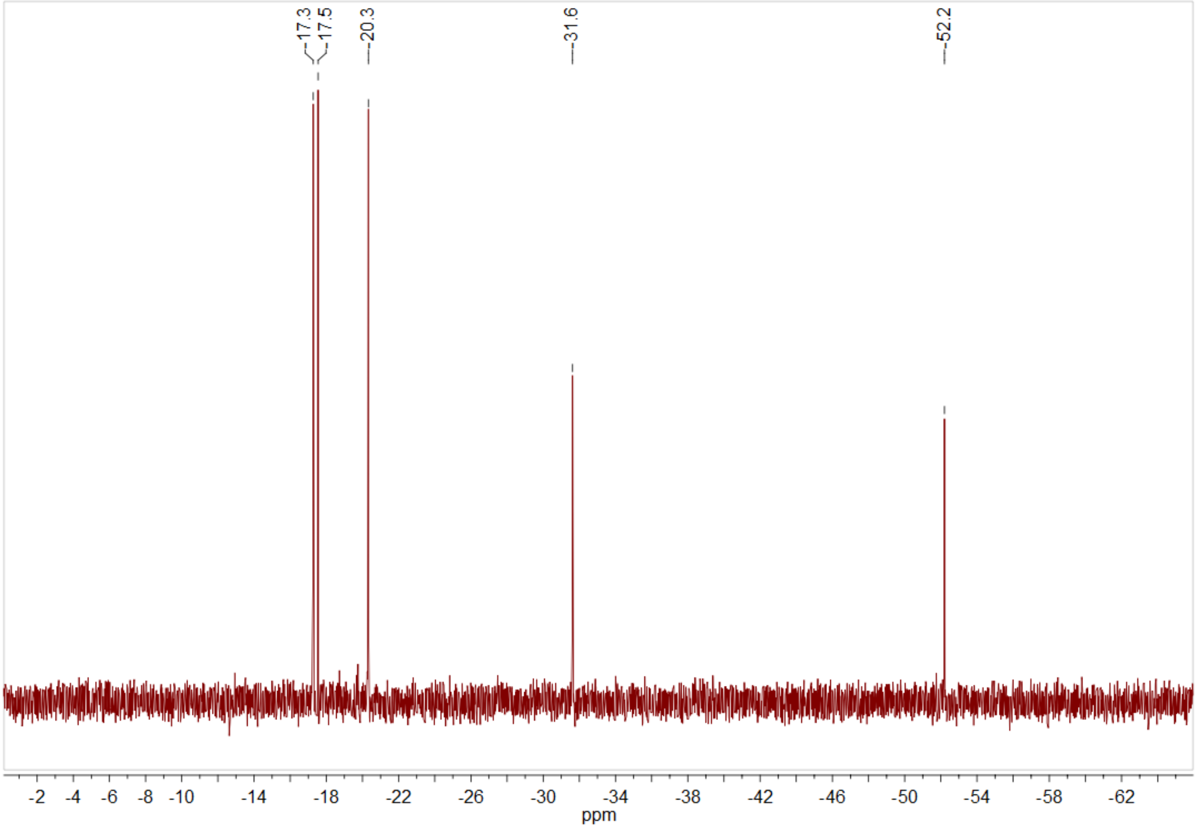

Figure S7. ^{1}H-NMR spectrum of **3** (300 MHz, C_6D_6 solution)

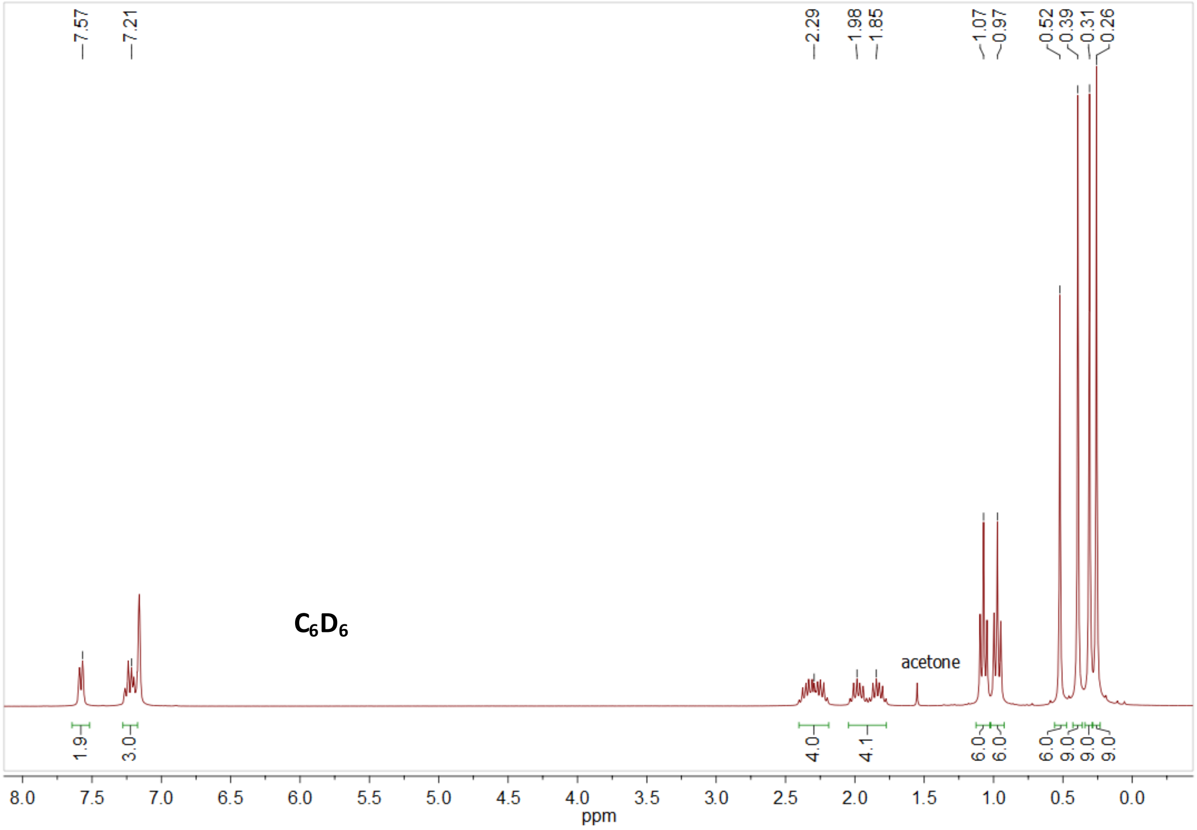

Figure S8. ^{13}C{^{1}H}-NMR spectrum of **3** (75 MHz, C_6D_6 solution)

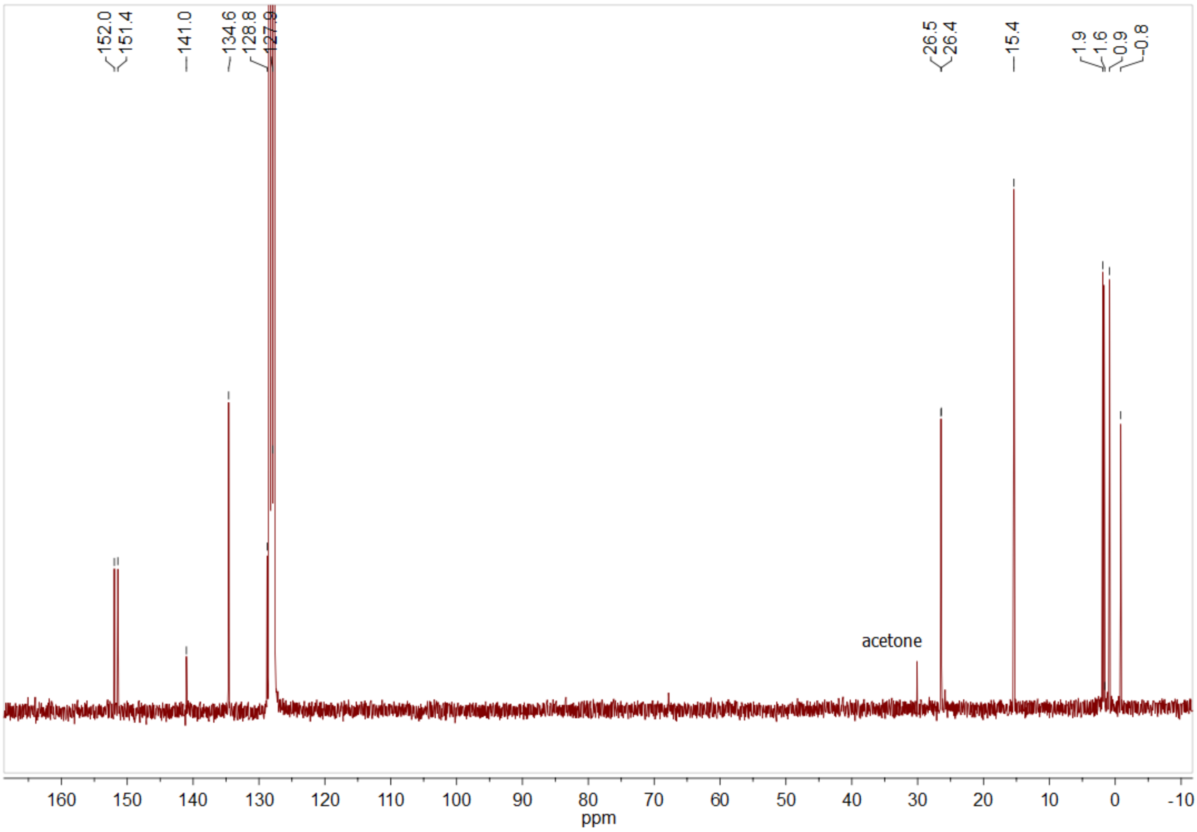

Figure S9. $^{29}Si\{^1H\}$-DEPT-NMR spectrum of **3** (60 MHz, C_6D_6 solution)

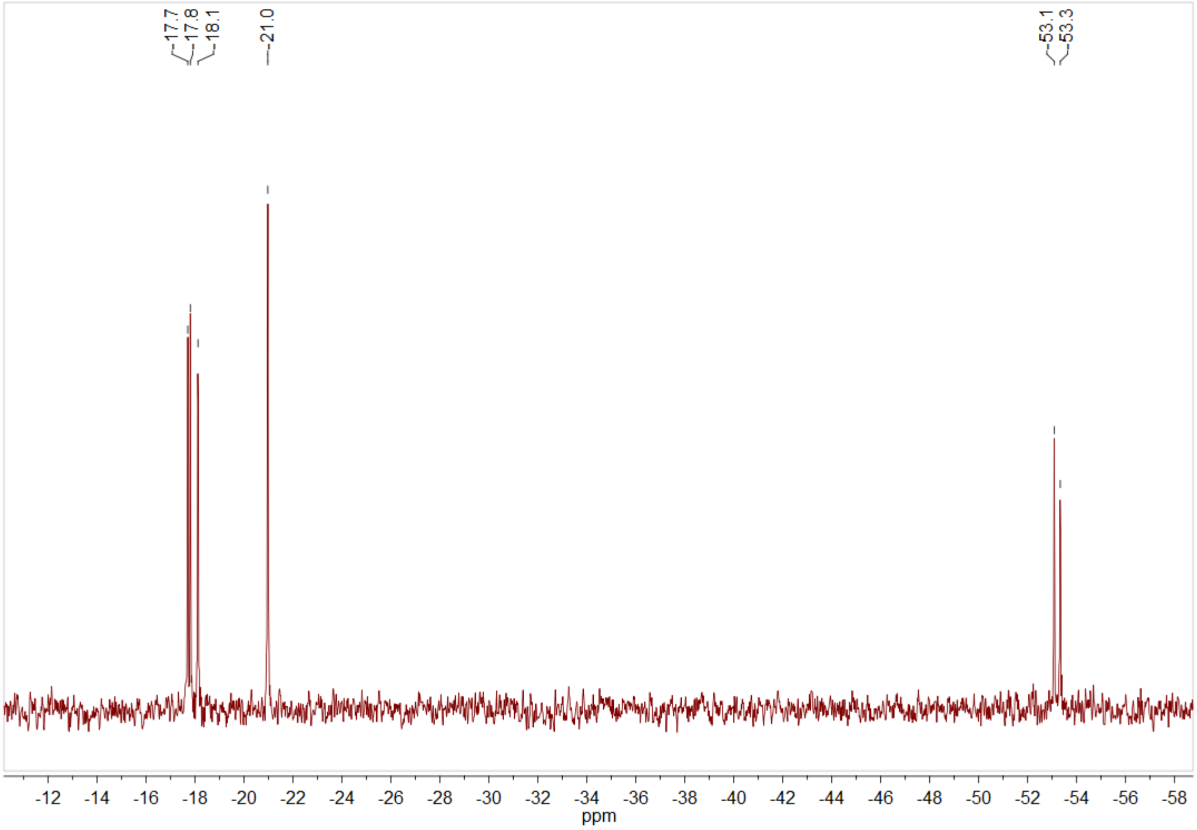

Figure S10. ^1H-NMR spectrum of **4** (300 MHz, C_6D_6 solution)

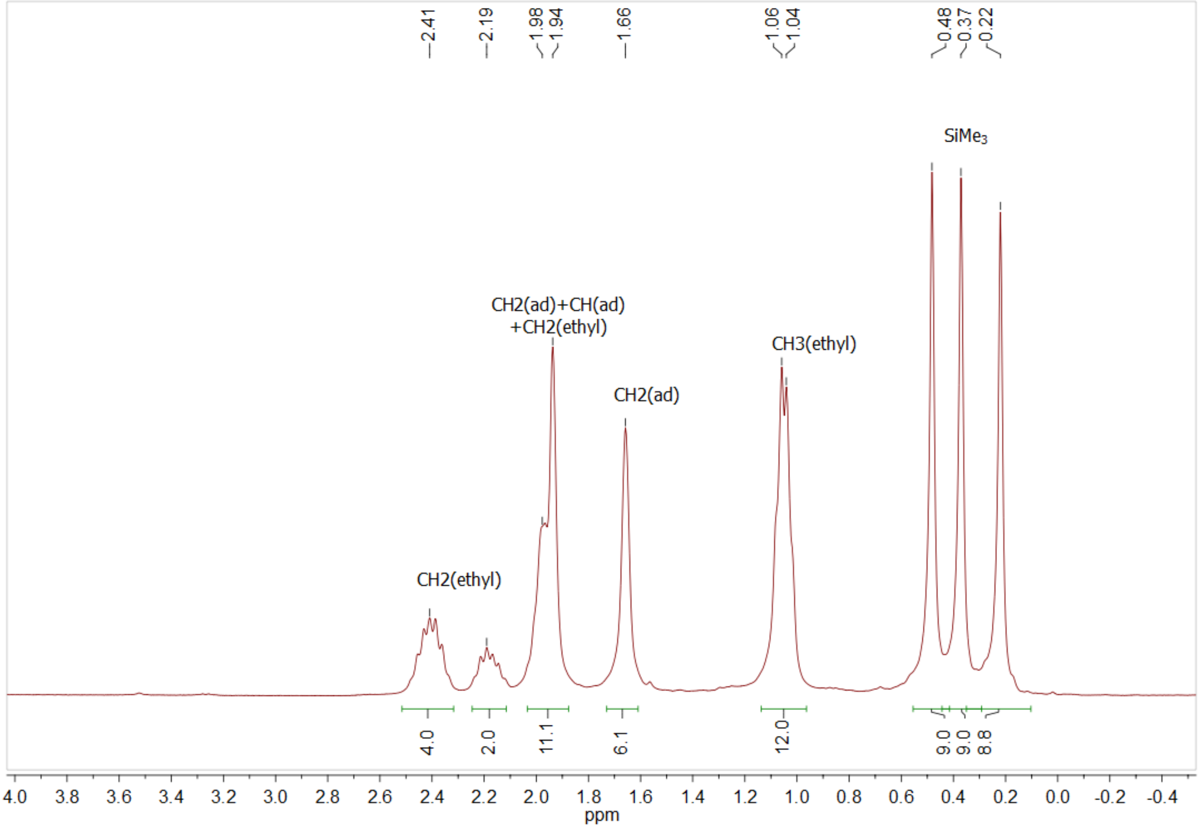

Figure S11. $^{13}C\{^{1}H\}$-NMR spectrum of **4** (75 MHz, C_6D_6 solution)

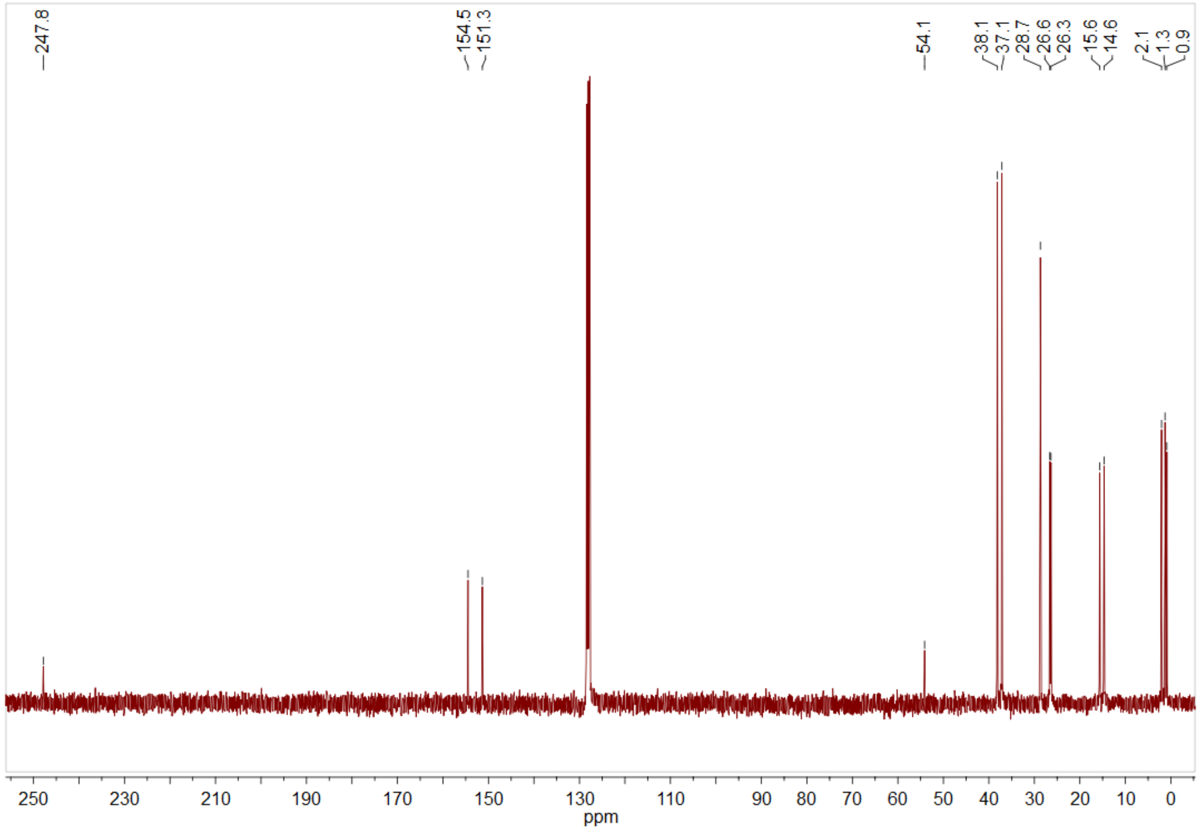

Figure S12. $^{29}Si\{^{1}H\}$-INEPT-NMR spectrum of **4** (60 MHz, C_6D_6 solution)

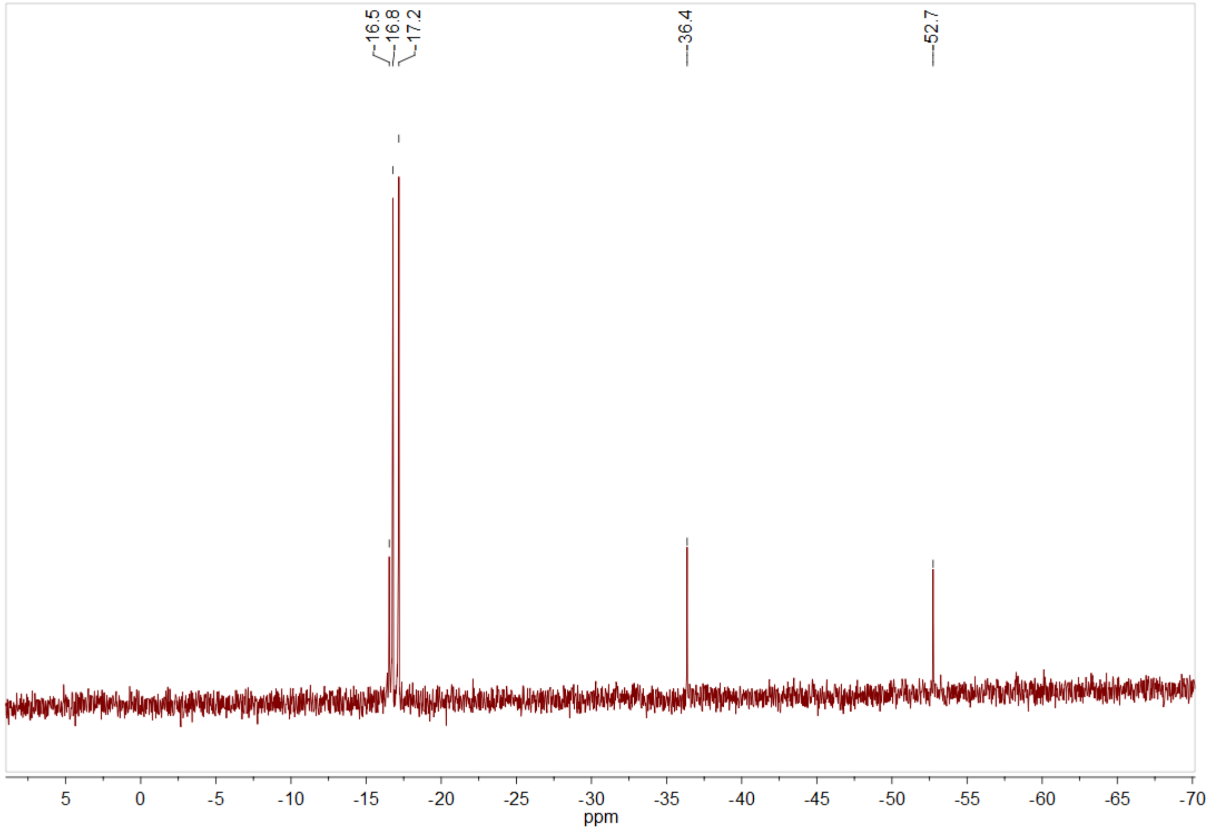

Figure S13. ^{1}H-NMR spectrum of **5** (300 MHz, C_6D_6 solution)

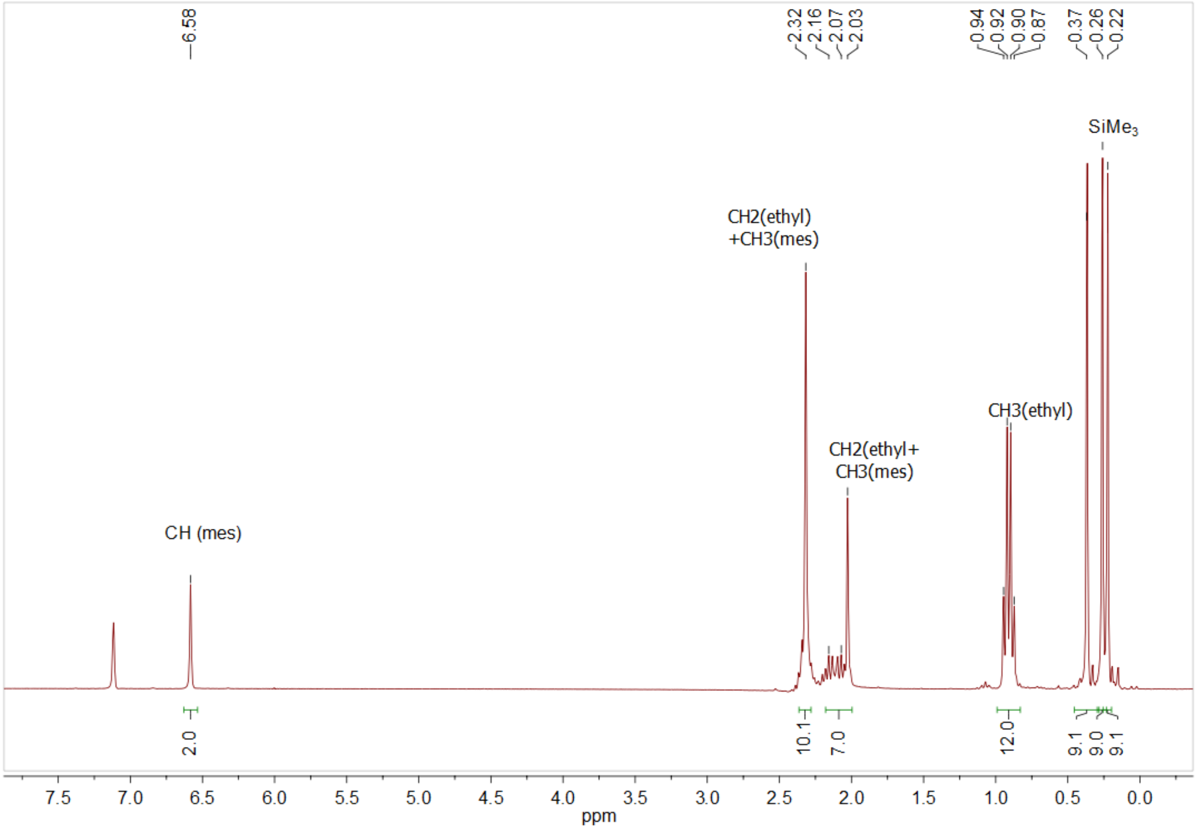

Figure S14. ^{13}C{^{1}H}-NMR spectrum of **5** (75 MHz, C_6D_6 solution)

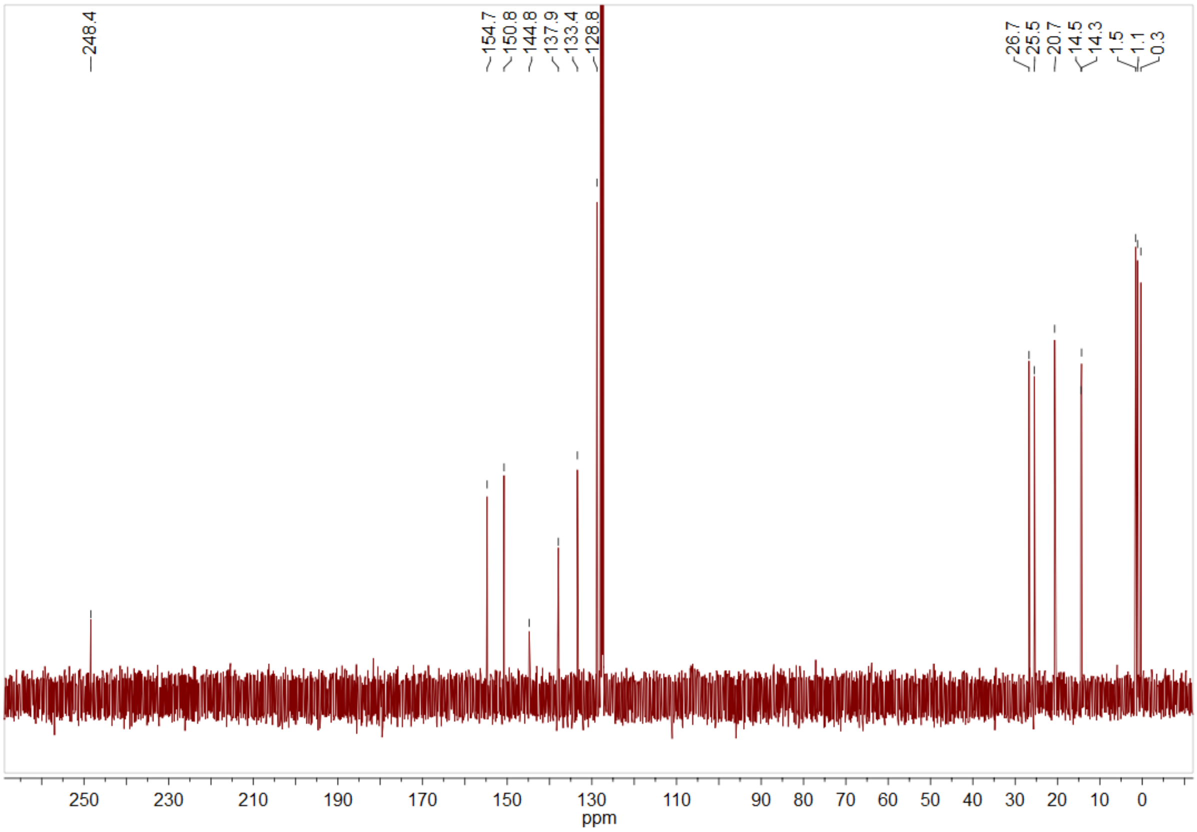

Figure S15. $^{29}Si\{^1H\}$-INEPT-NMR spectrum of **5** (60 MHz, C_6D_6 solution)

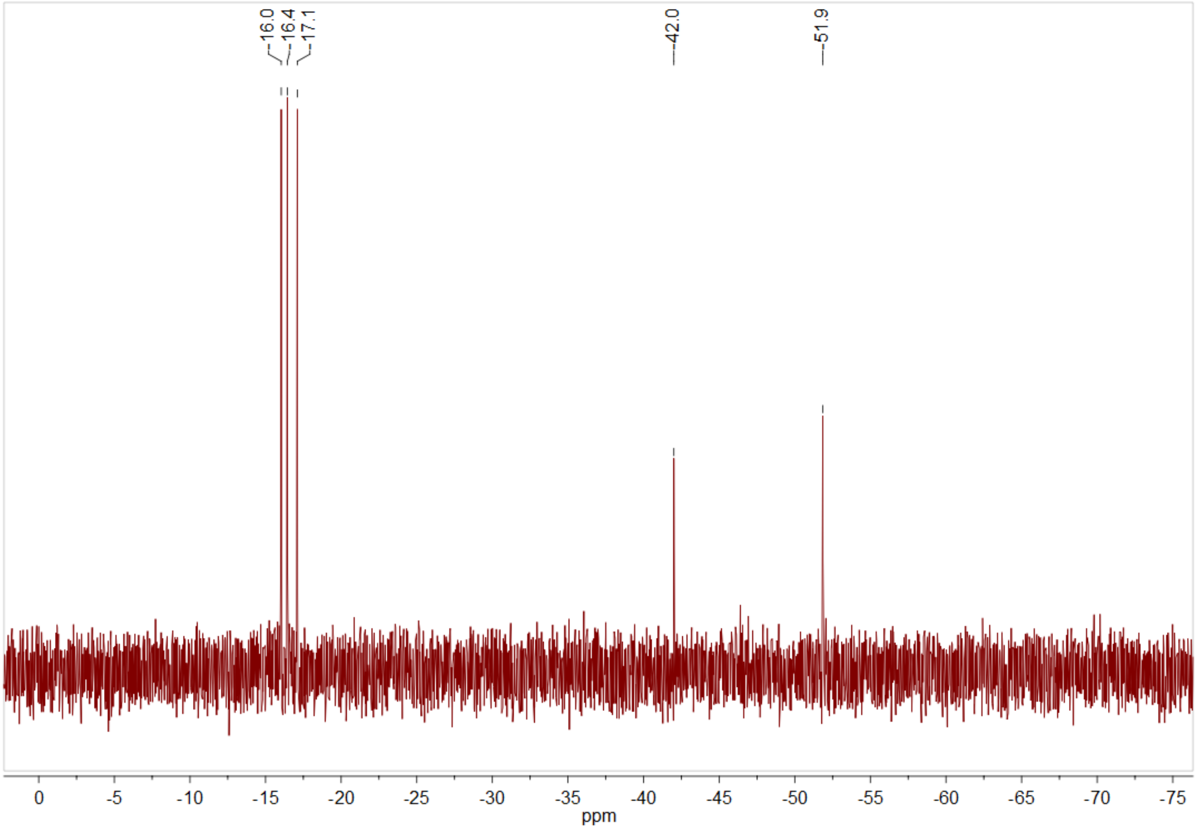

Figure S16. ^1H-NMR spectrum of **4** after irradiation in neat d_6-benzene solution (c ≈ 0.25 M) with λ >300 nm light for 0, 30 and 90 minutes.

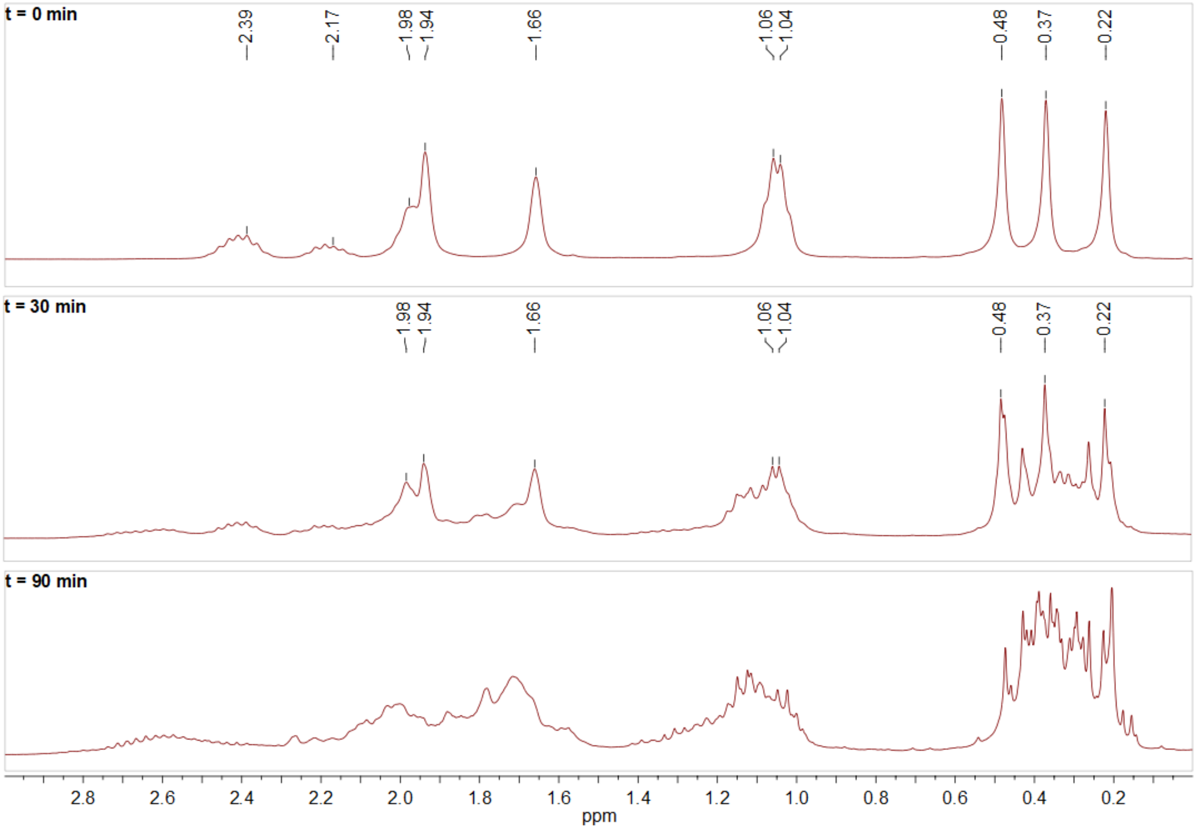

Figure S17. ^{13}C-NMR spectrum of **4** after irradiation in neat d_6-benzene solution (c ≈ 0.25 M) with λ >300 nm light for 0, 30 and 90 minutes.

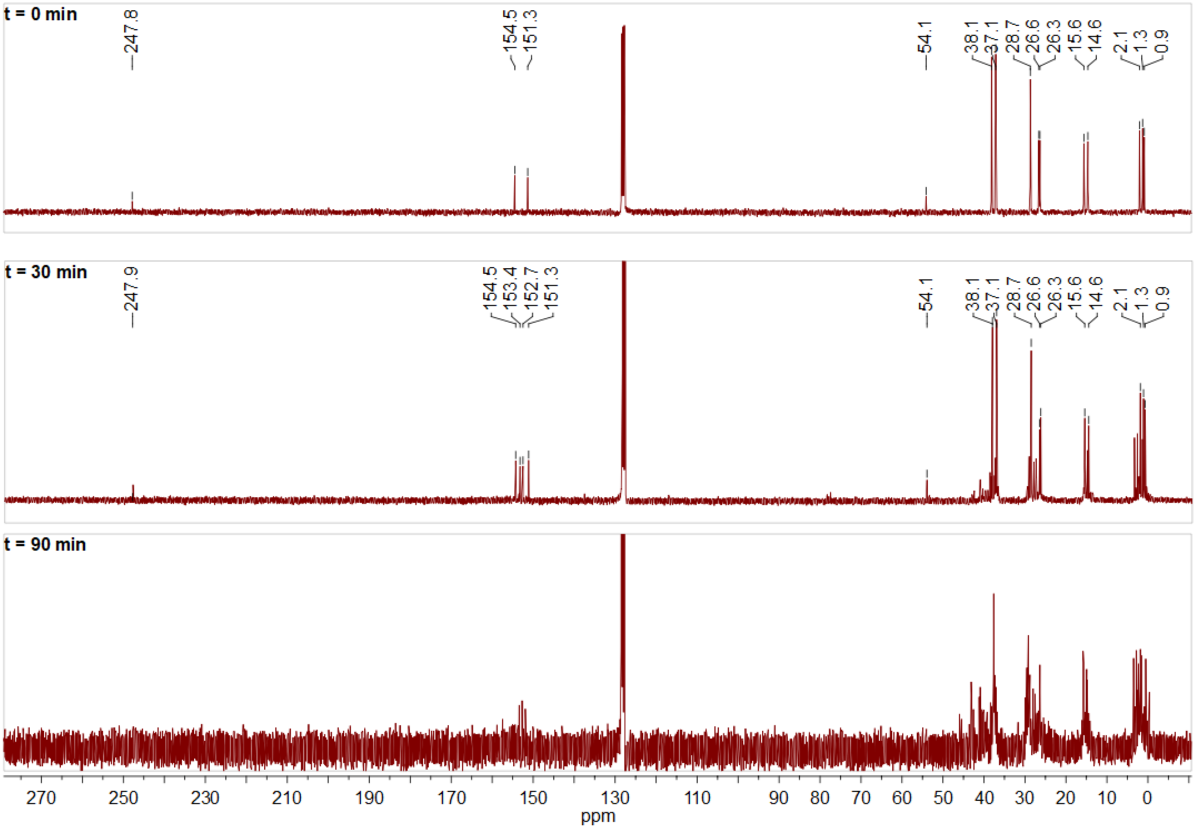

Figure S18. ^{1}H-NMR spectrum of **6** (300 MHz, C_6D_6 solution)

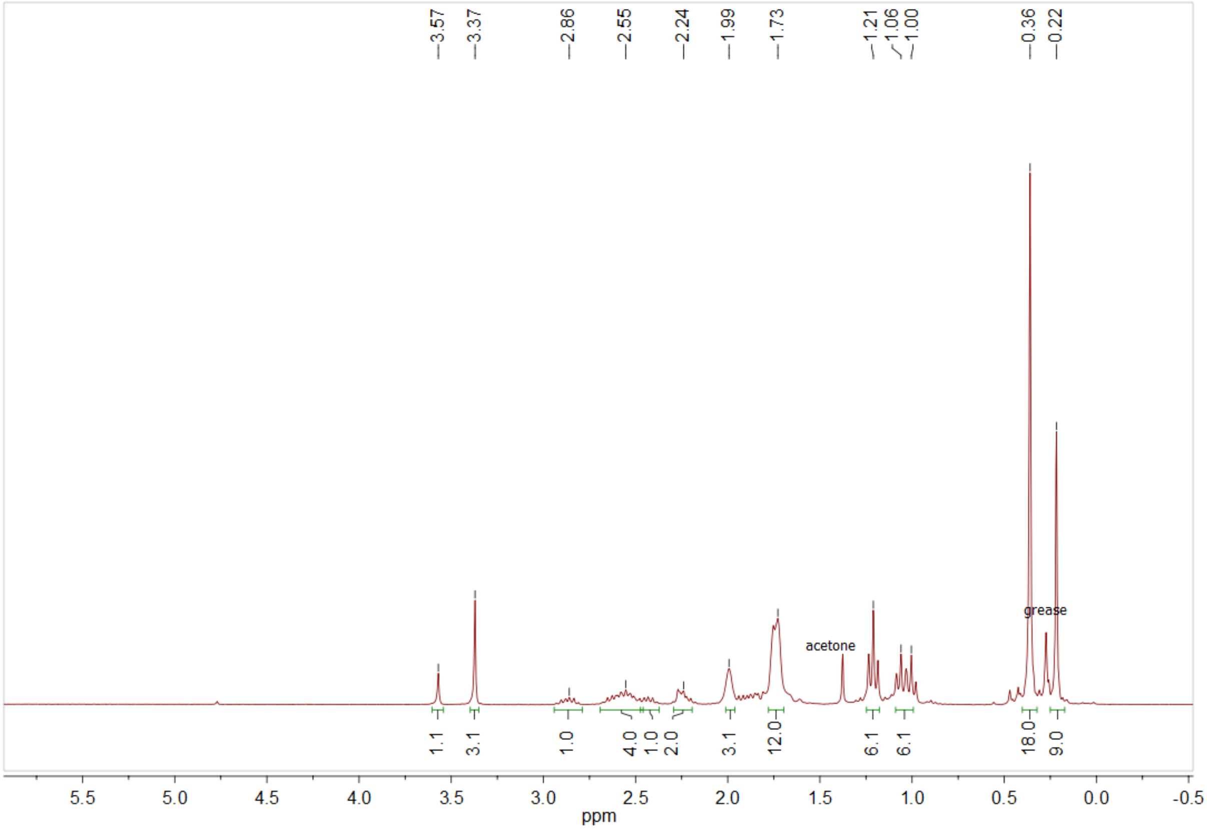

Figure S19. $^{13}C\{^{1}H\}$-NMR spectrum of **6** (75 MHz, C_6D_6 solution)

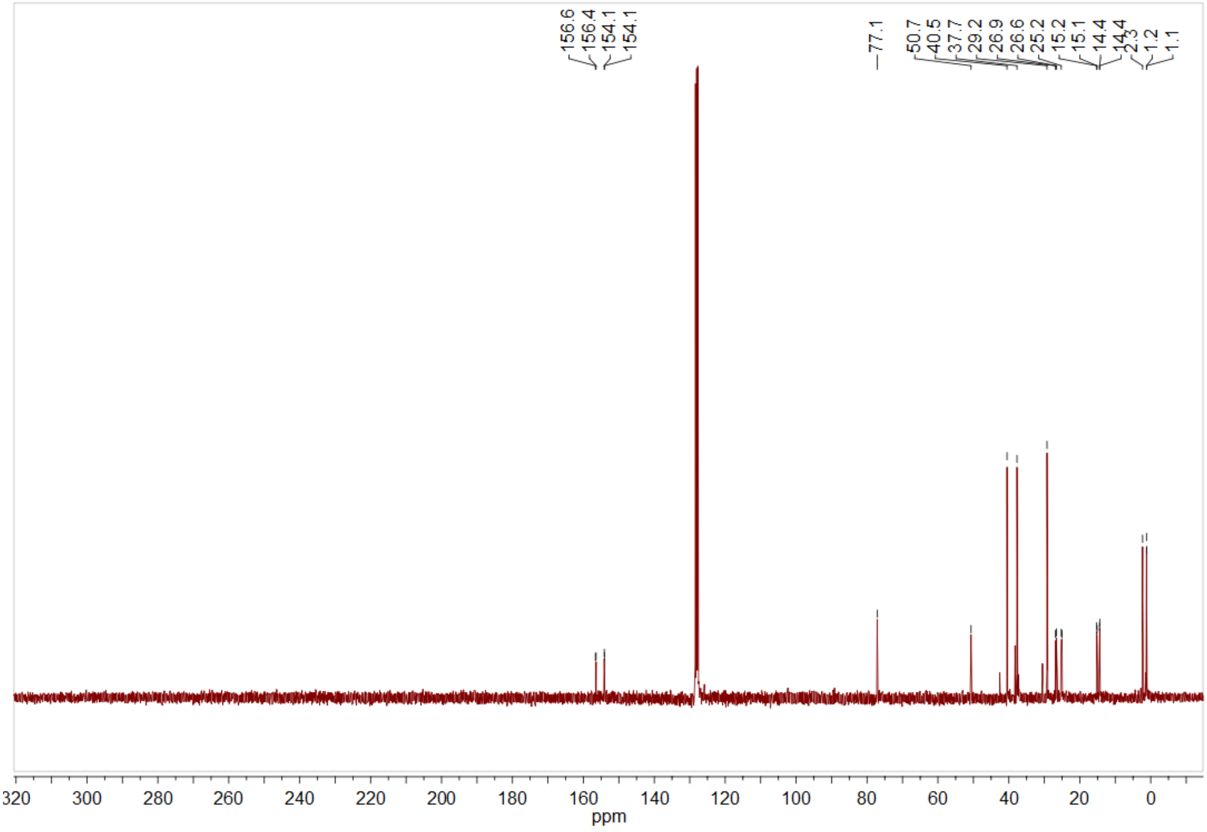

Figure S20. $^{29}Si\{^{1}H\}$-INEPT-NMR spectrum of **6** (60 MHz, C_6D_6 solution)

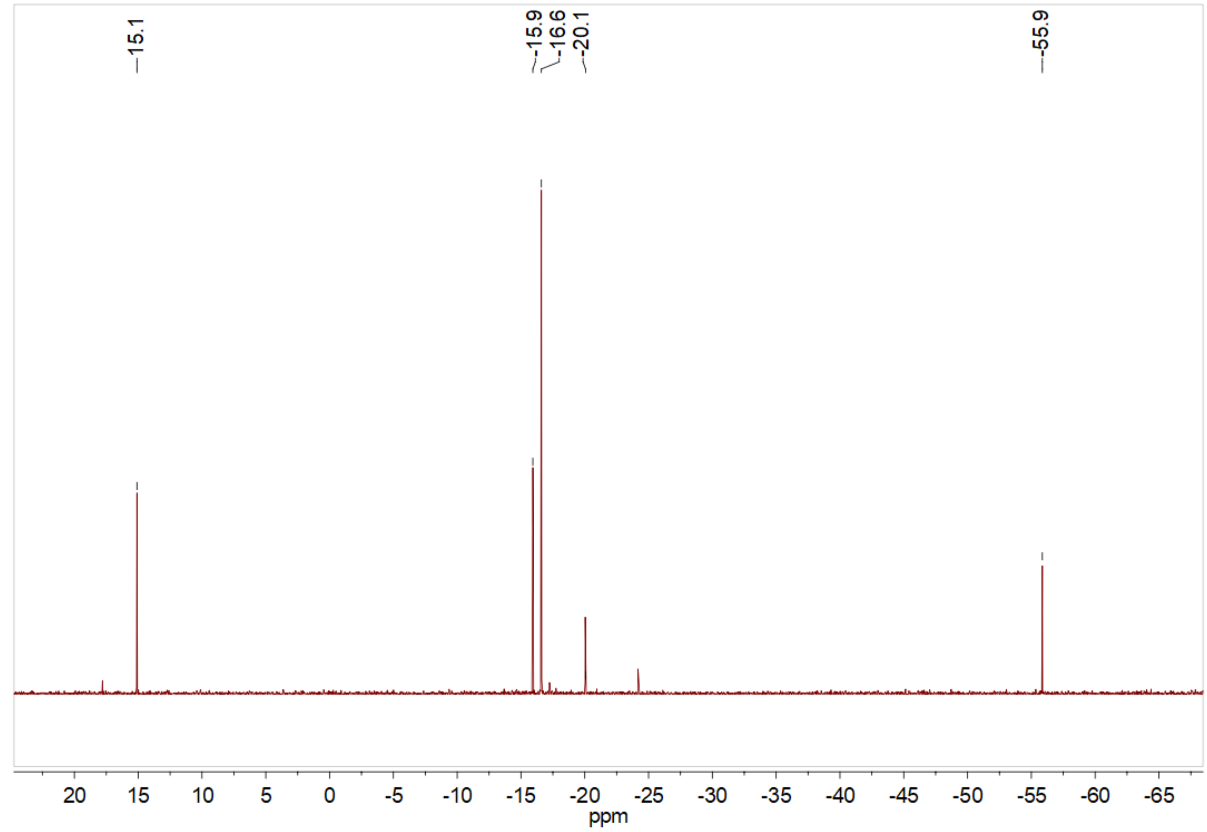

Figure S21. ^{1}H-NMR spectrum of **7** (300 MHz, C_6D_6 solution)

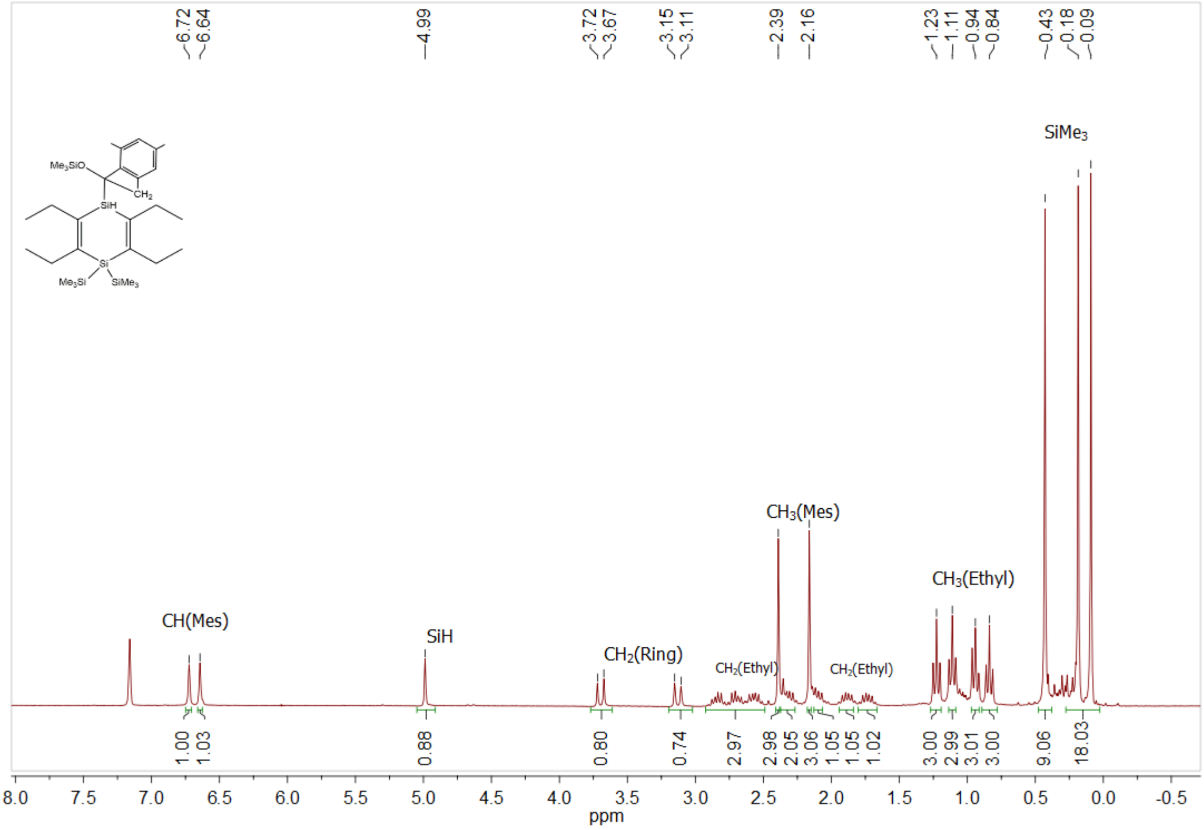

Figure S22. ^{13}C{^{1}H}-NMR spectrum of **7** (75 MHz, C_6D_6 solution)

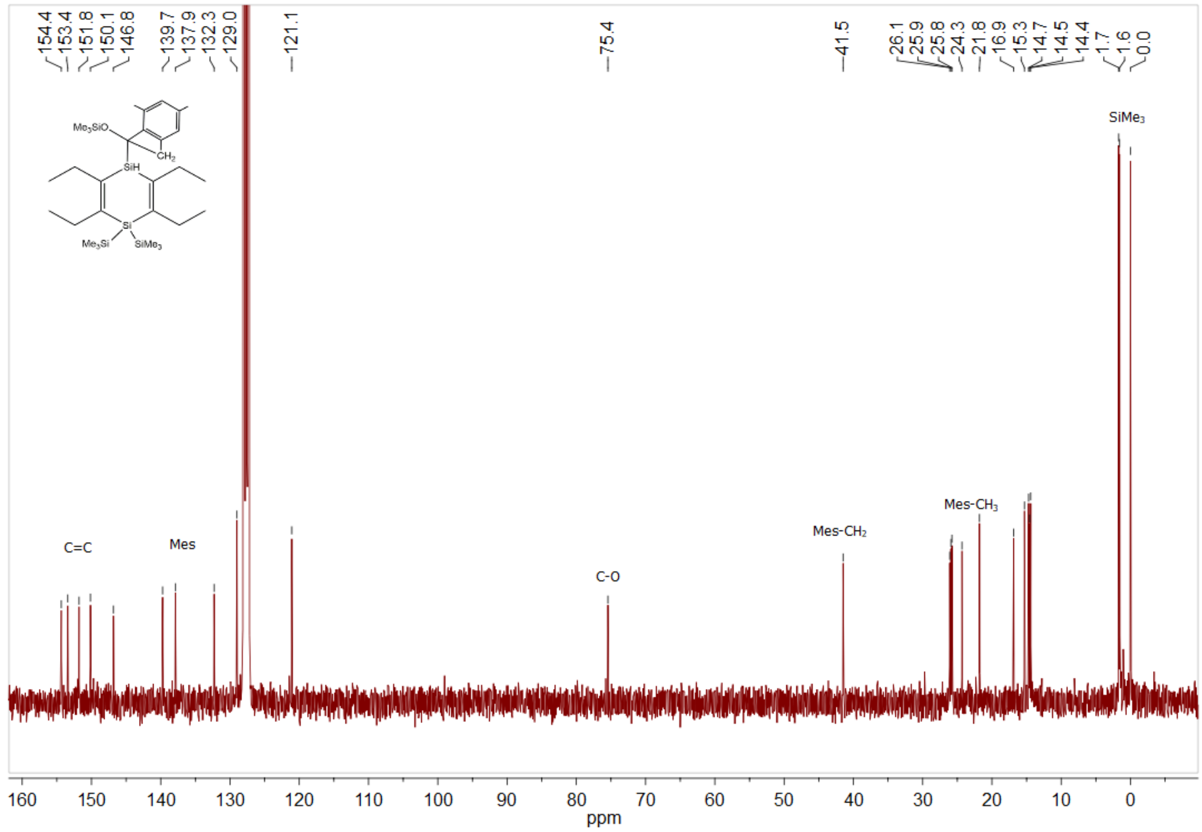

Figure S23. $^{29}Si\{^1H\}$-and $^{29}Si\{^1H\}$ DEPT-NMR spectra of **7** (60 MHz, C_6D_6 solution)

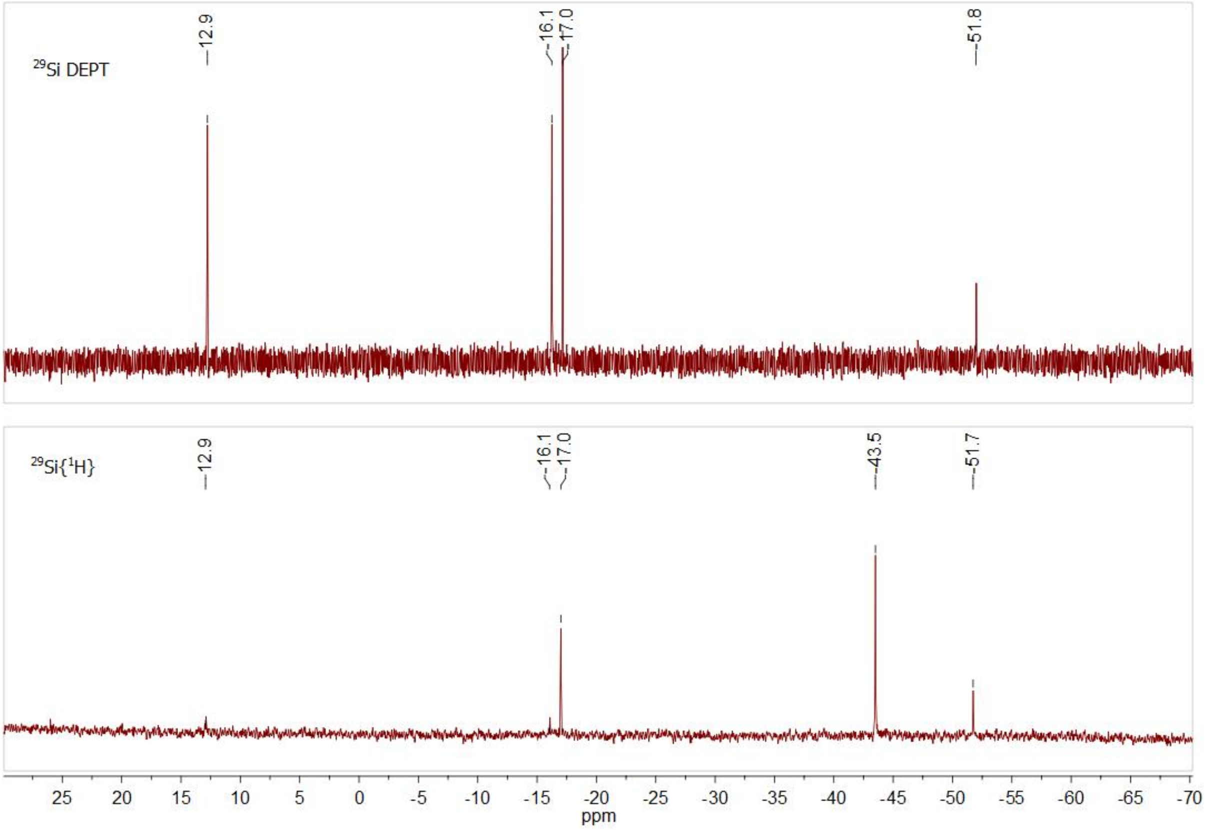

Figure S24. ^1H-NMR spectrum of **8a** (300 MHz, C_6D_6 solution)

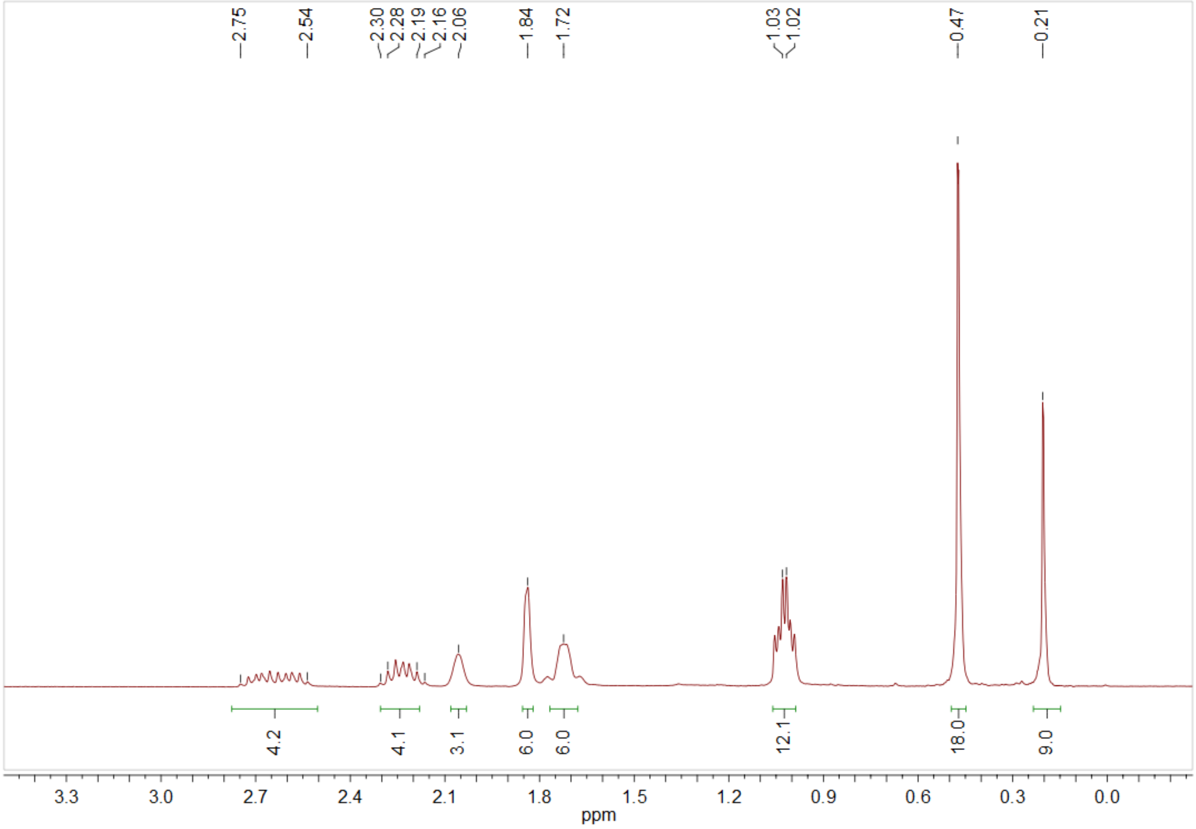

Figure S25. $^{13}C\{^{1}H\}$-NMR spectrum of **8a** (75 MHz, C_6D_6 solution)

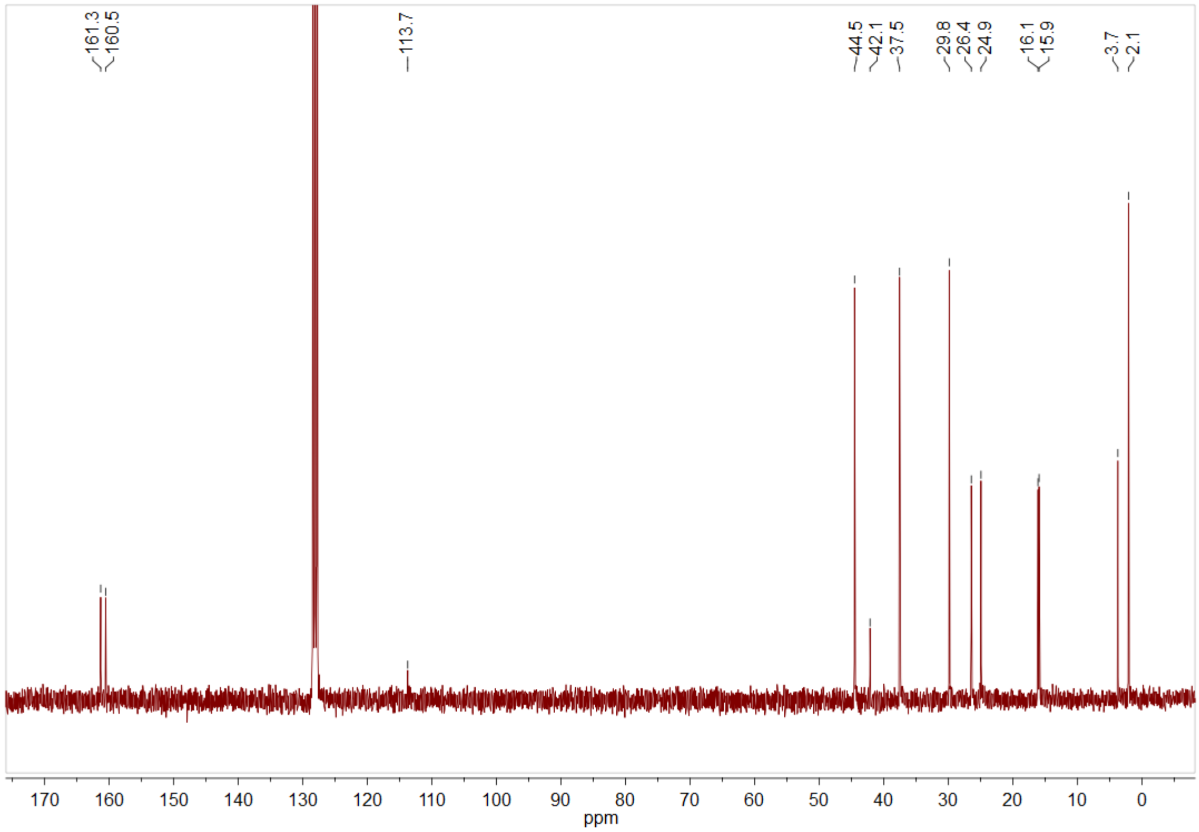

Figure S26. $^{29}Si\{^{1}H\}$-INEPT-NMR spectrum of **8a** (60 MHz, C_6D_6 solution)

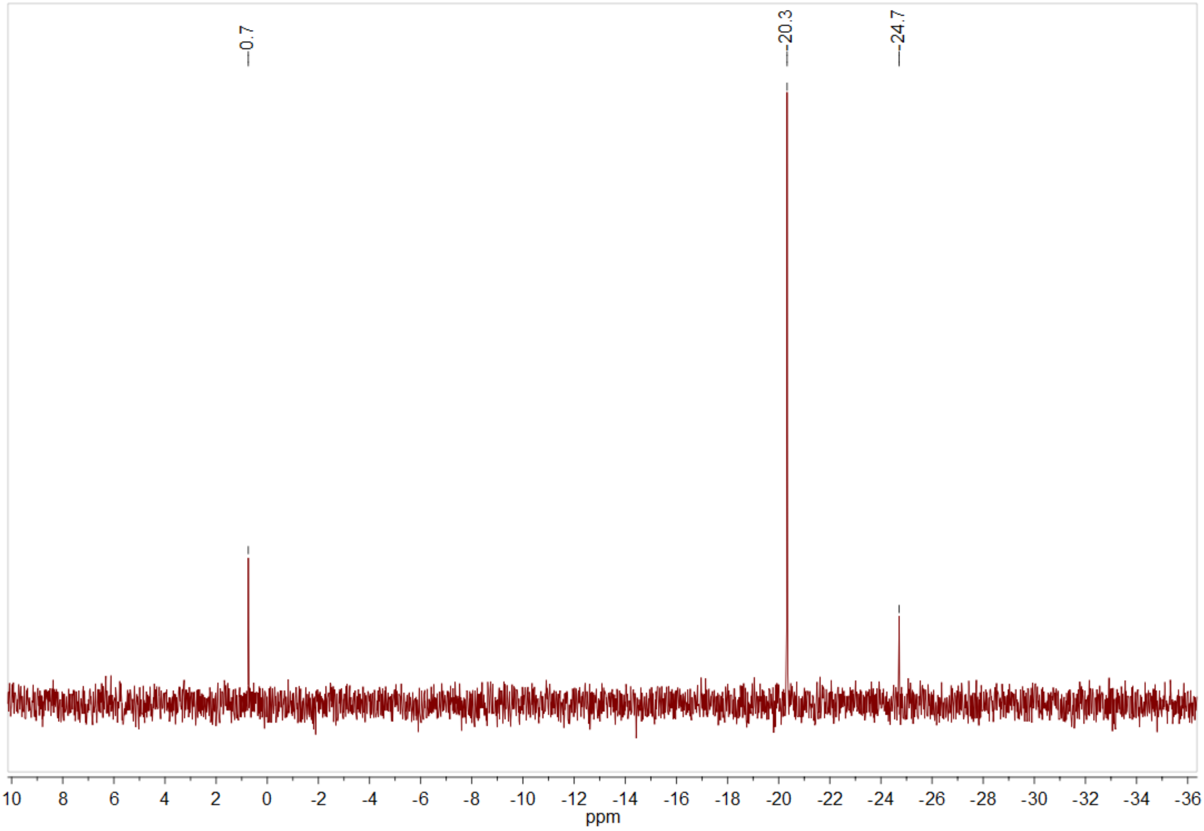

Figure S27. ^{1}H-NMR spectrum of **8b** (300 MHz, DME solution)

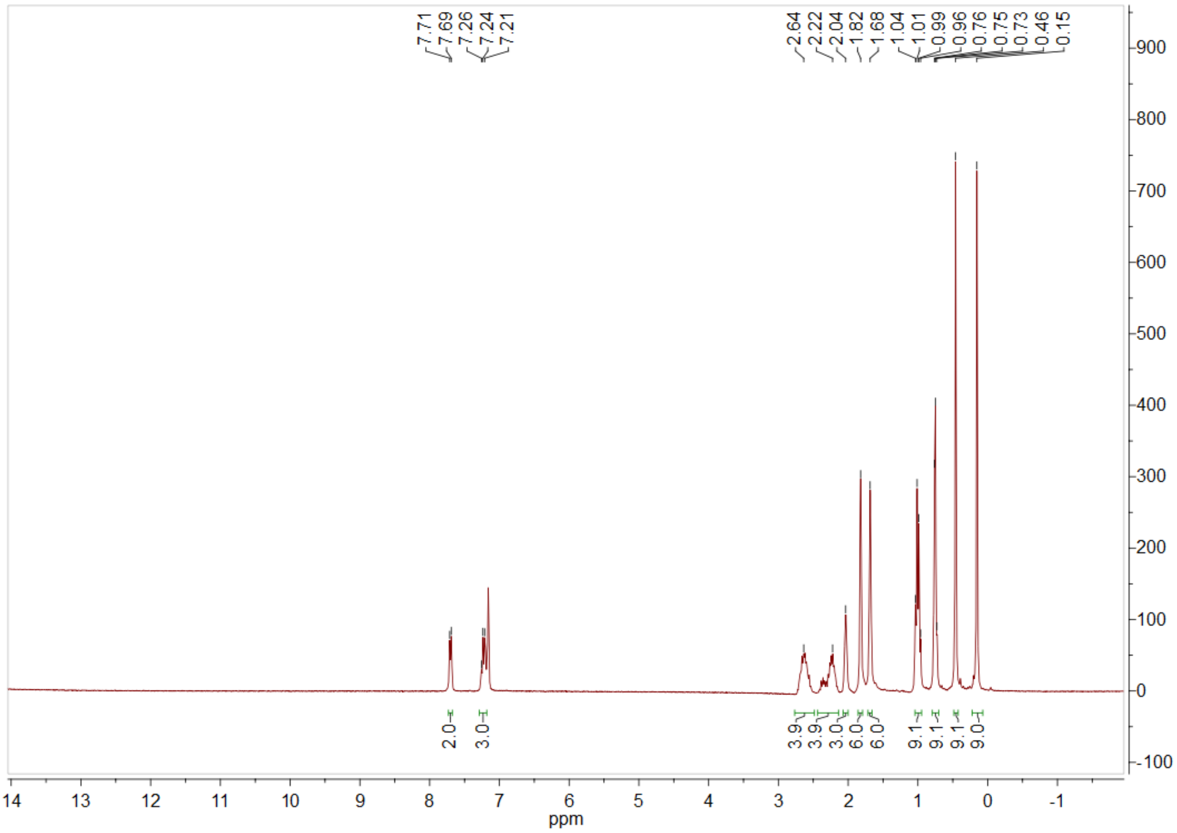

Figure S28 ^{13}C{^{1}H}-NMR spectrum of **8b** (75 MHz, DME solution)

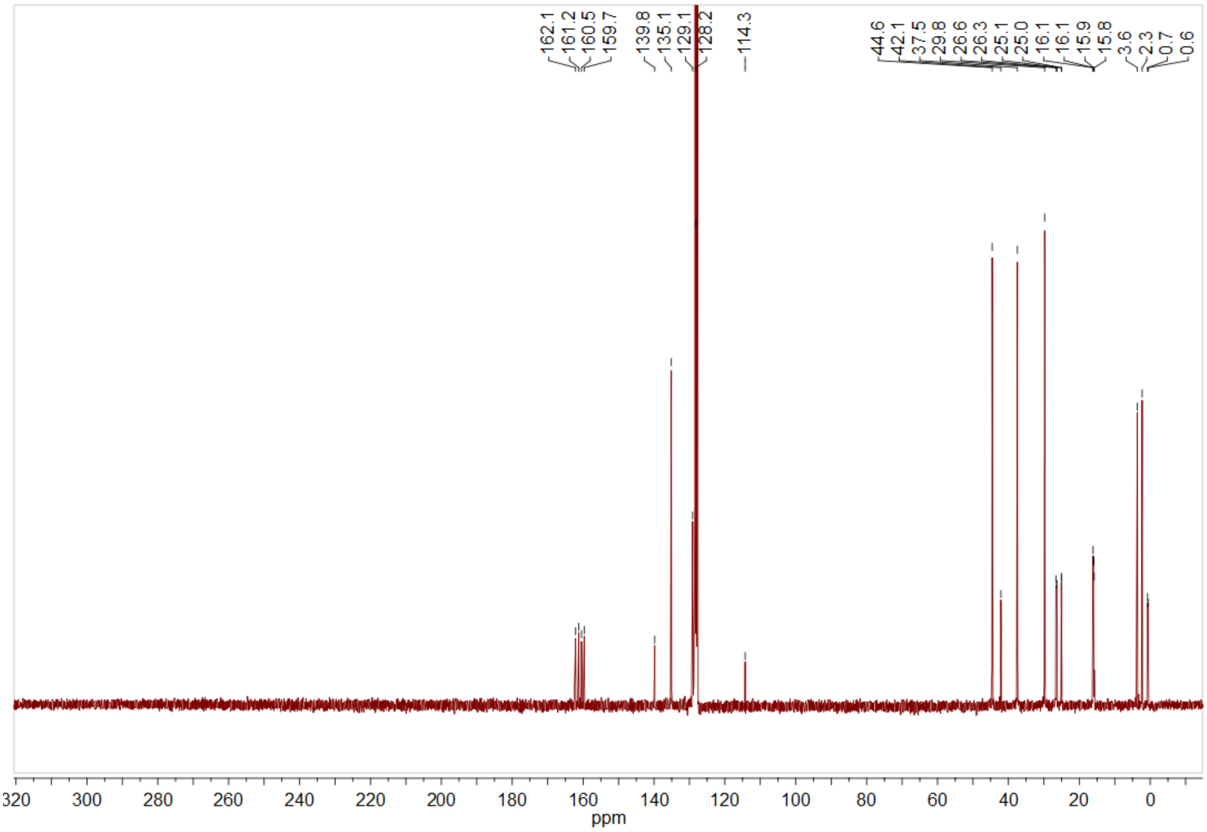

Figure S29. $^{29}Si\{^{1}H\}$-INEPT-NMR spectrum of **8b** (60 MHz, C_6D_6 solution)

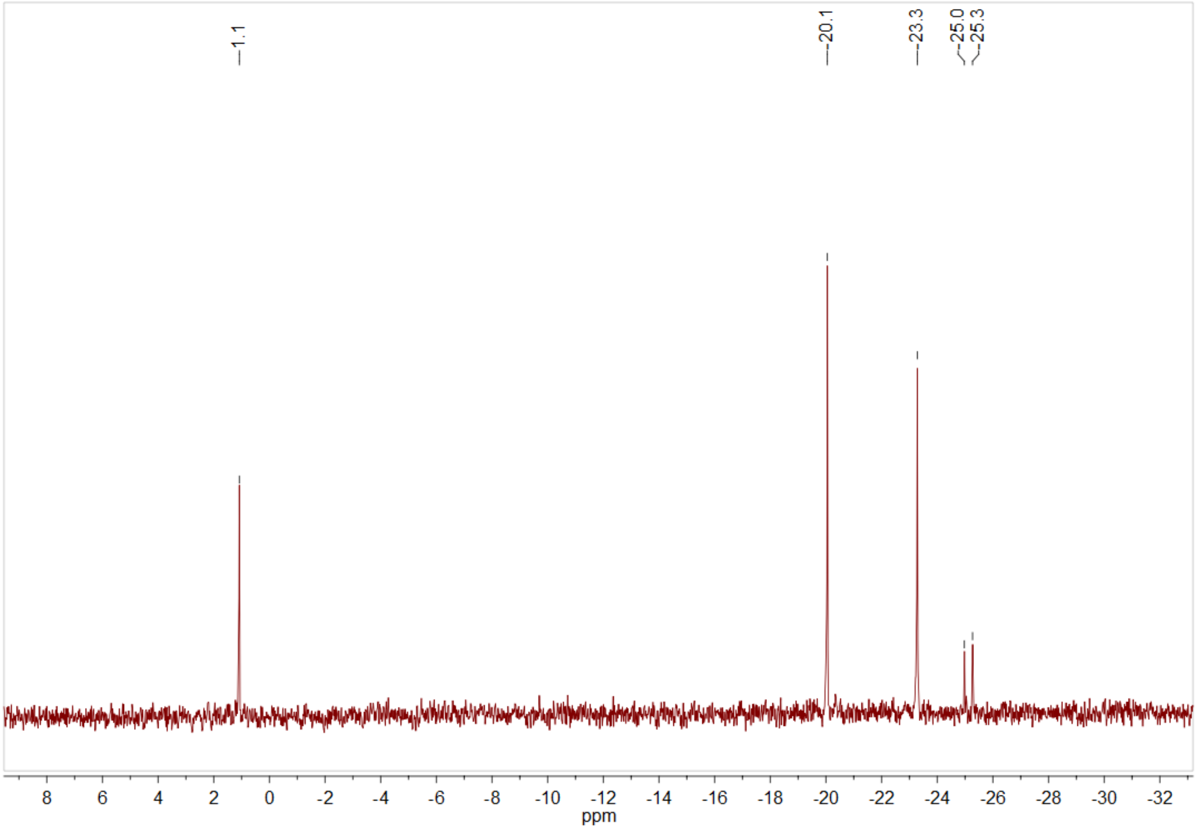

Figure S30. ^{1}H-NMR spectrum of **9** (300 MHz, C_6D_6 solution)

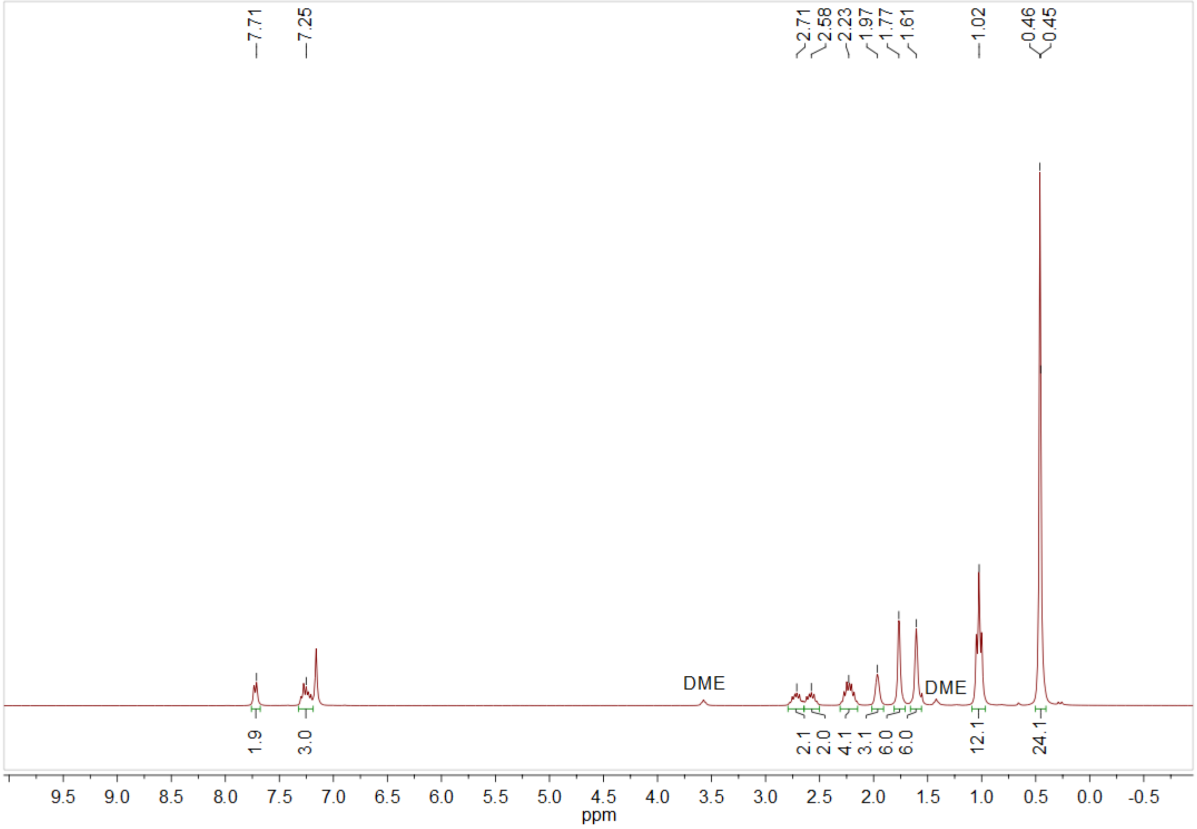

Figure S31. $^{13}C\{^1H\}$-NMR spectrum of **9** (75 MHz, C_6D_6 solution)

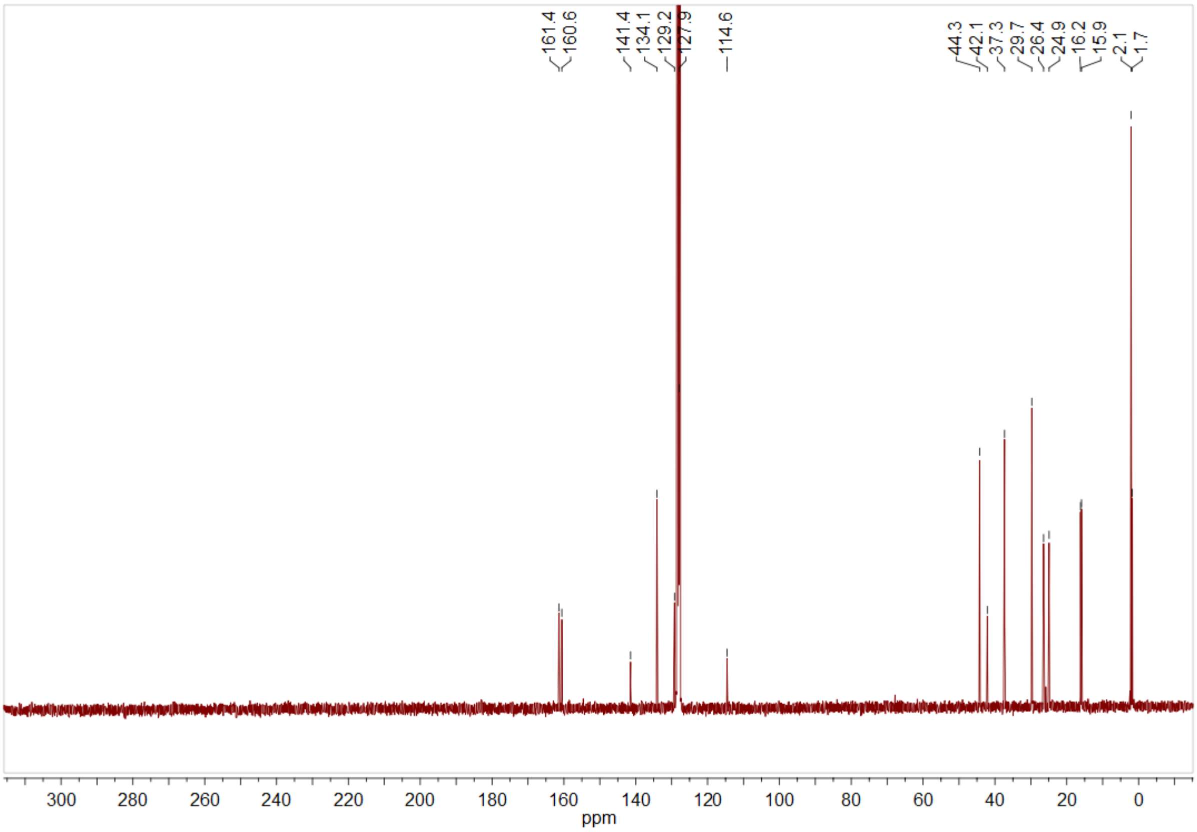

Figure S32. $^{29}Si\{^1H\}$-INEPT-NMR spectrum of **9** (60 MHz, C_6D_6 solution)

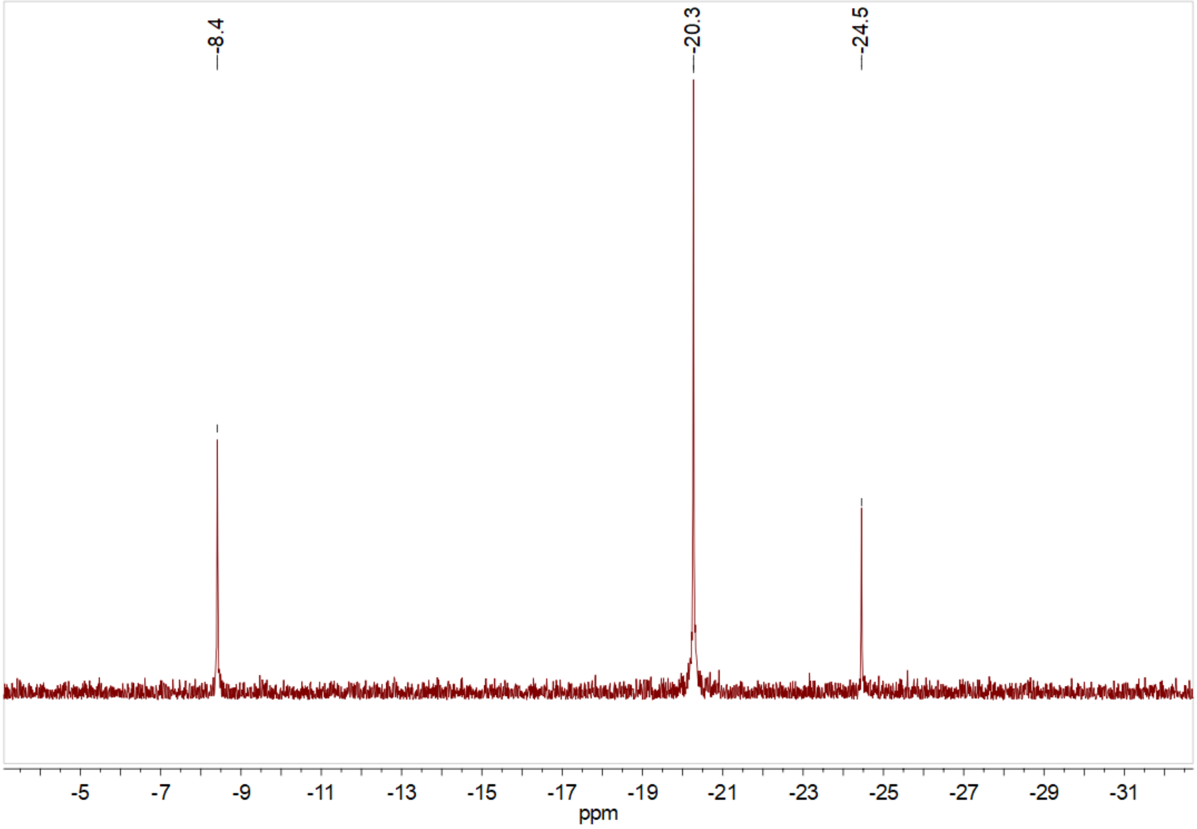

Figure S33. Molecular structure of **2**. All hydrogen atoms are omitted for clarity. Thermal ellipsoids are set at 30% probability level. Selected bond lengths [pm] and bond and torsional angles [deg] with estimated standard deviations.

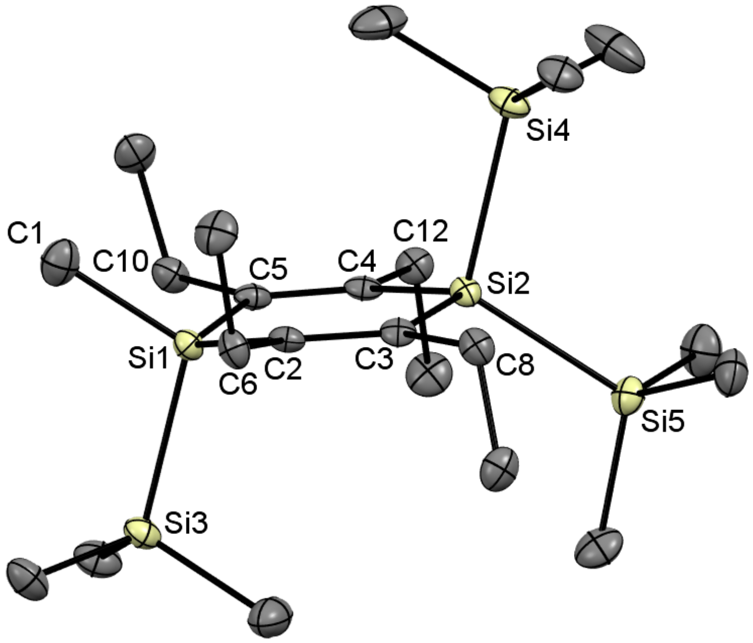

bond distance [pm]		bond angle [°]		torsion angle [°]	
Si(1)–Si(3)	236.0(1)	Si(1)–C(2)–C(3)	123.0(1)	Si(1)–C(2)–C(3)–Si(2)	15.7(2)
Si(2)–Si(4)	236.7(1)	C(2)–C(3)–Si(2)	122.9(1)	C(2)–C(3)–Si(2)–C(4)	−10.3(2)
Si(2)–Si(5)	236.7(1)	C(3)–Si(2)–C(4)	112.3(1)	C(3)–Si(2)–C(4)–C(5)	9.5(2)
Si(1)–C(1)	188.3(2)	Si(2)–C(4)–C(5)	123.2(1)	Si(2)–C(4)–C(5)–Si(1)	−14.2(2)
Si(1)–C(2)	186.8(2)	C(4)–C(5)–Si(1)	122.8(2)	C(4)–C(5)–Si(1)–C(2)	16.7(2)
Si(1)–C(5)	186.9(2)	C(5)–Si(1)–C(2)	112.6(1)	C(5)–Si(1)–C(2)–C(3)	−17.6(2)
Si(2)–C(3)	188.3(2)				
Si(2)–C(4)	188.5(2)				
C(2)–C(3)	135.3(3)				
C(4)–C(5)	135.2(3)				

Figure S34. Molecular structure of **3**. All hydrogen atoms are omitted for clarity. Thermal ellipsoids are set at 30% probability level. All hydrogen atoms are omitted for clarity. Thermal ellipsoids are set at 30% probability level. Selected bond lengths [pm] and bond and torsional angles [deg] with estimated standard deviations.

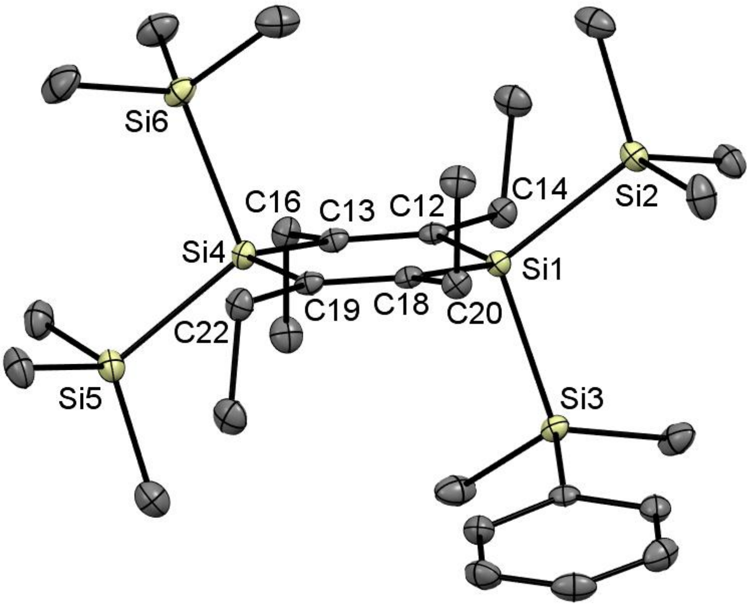

bond distance [pm]		bond angle [°]		torsion angle [°]	
Si(1)–Si(2)	237.3(1)	Si(1)–C(12)–C(13)	123.0(1)	Si(1)–C(12)–C(13)–Si(4)	13.0(2)
Si(1)–Si(3)	237.4(1)	C(12)–C(13)–Si(4)	123.5(1)	C(12)–C(13)–Si(4)–C(19)	−10.9(2)
Si(4)–Si(5)	237.0(1)	C(13)–Si(4)–C(19)	112.3(1)	C(13)–Si(4)–C(19)–C(18)	10.4(2)
Si(4)–Si(6)	237.2(1)	Si(4)–C(19)–C(18)	123.6(1)	Si(4)–C(19)–C(18)–Si(1)	−12.2(2)
Si(1)–C(12)	187.9(2)	C(19)–C(18)–Si(1)	123.0(1)	C(19)–C(18)–Si(1)–C(12)	11.8(2)
Si(1)–C(18)	189.3(2)	C(18)–Si(1)–C(12)	112.5(1)	C(18)–Si(1)–C(12)–C(13)	−12.3(2)
Si(4)–C(13)	189.0(2)				
Si(4)–C(19)	187.9(2)				
C(12)–C(13)	135.0(2)				
C(19)–C(18)	134.9(2)				

Figure S35. Most stable DFT CPCM (THF) B3LYP-GD3/6-31+G(d) calculated conformers and relative energies of **1-K**.

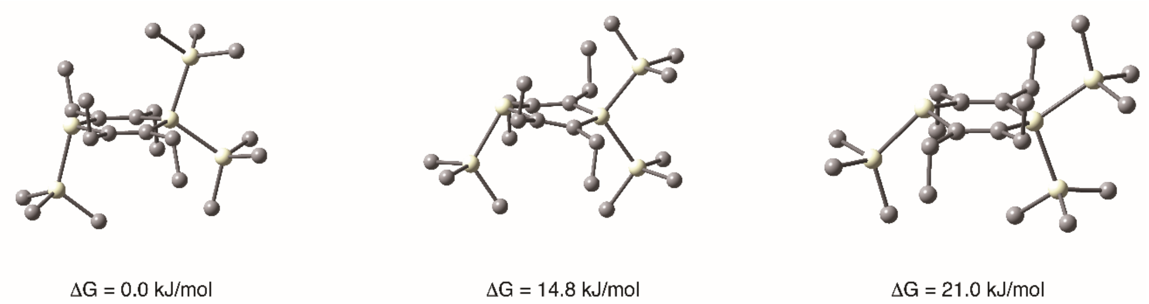

Figure S36. DFT CPCM (THF) B3LYP-GD3/6-31+G(d) calculated structure of the most stable conformer of **4**. All hydrogen atoms are omitted for clarity. Selected calculated bond lengths [pm] and bond and torsional angles [deg].

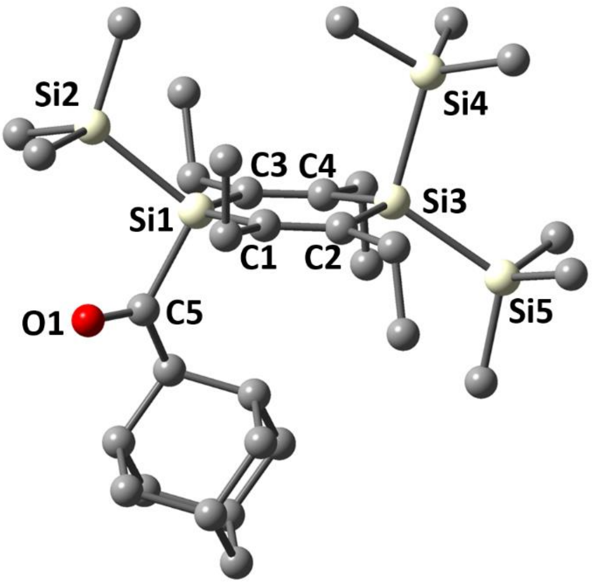

bond distance [pm]		bond angle [°]		torsion angle [°]	
Si(1)–Si(2)	238.9	Si(1)–C(1)–C(2)	123.2	Si(1)–C(1)–C(2)–Si(3)	10.6
Si(3)–Si(4)	238.8	C(1)–C(2)–Si(3)	123.3	C(1)–C(2)–Si(3)–C(4)	−14.3
Si(3)–Si(5)	238.0	C(2)–Si(3)–C(4)	112.1	C(2)–Si(3)–C(4)–C(3)	13.4
Si(1)–C(1)	190.2	Si(3)–C(4)–C(3)	123.1	Si(3)–C(4)–C(3)–Si(1)	−9.1
Si(1)–C(3)	190.0	C(4)–C(3)–Si(1)	123.4	C(4)–C(3)–Si(1)–C(1)	3.5
Si(3)–C(2)	189.0	C(3)–Si(1)–C(1)	113.2	C(3)–Si(1)–C(1)–C(2)	−4.3
Si(3)–C(4)	189.0				
Si(1)–C(5)	197.5				
C(1)–C(2)	135.9				
C(3)–C(4)	135.8				
Si(1)–C(5)	135.8				
C(5)–O(1)	123.2				

Figure S37. DFT CPCM (THF) B3LYP-GD3/6-31+G(d) calculated structure of the most stable conformer of **5**. All hydrogen atoms are omitted for clarity. Selected calculated bond lengths [pm] and bond and torsional angles [deg].

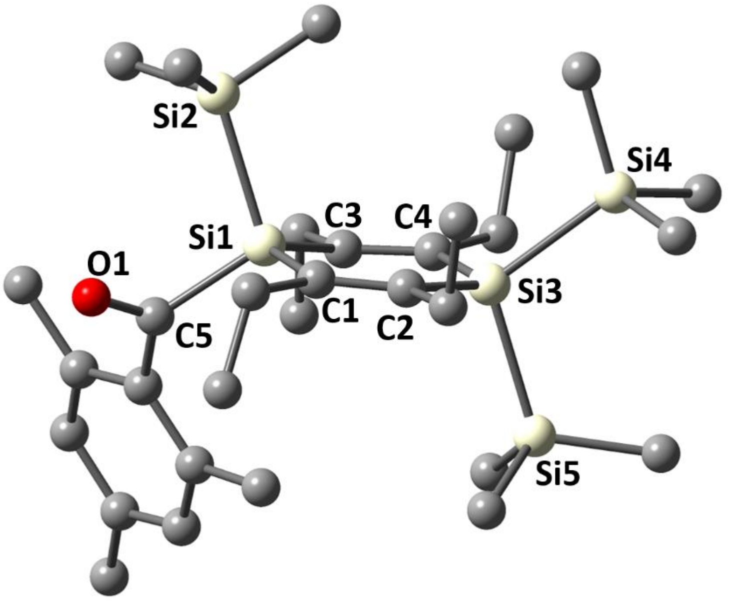

bond distance [pm]		bond angle [°]		torsion angle [°]	
Si(1)–Si(2)	237.5	Si(1)–C(1)–C(2)	121.9	Si(1)–C(1)–C(2)–Si(3)	−9.3
Si(3)–Si(4)	238.8	C(1)–C(2)–Si(3)	123.8	C(1)–C(2)–Si(3)–C(4)	7.4
Si(3)–Si(5)	239.3	C(2)–Si(3)–C(4)	112.3	C(2)–Si(3)–C(4)–C(3)	−12.1
Si(1)–C(1)	188.8	Si(3)–C(4)–C(3)	123.4	Si(3)–C(4)–C(3)–Si(1)	17.8
Si(1)–C(3)	188.2	C(4)–C(3)–Si(1)	121.4	C(4)–C(3)–Si(1)–C(1)	−17.4
Si(3)–C(2)	190.4	C(3)–Si(1)–C(1)	114.4	C(3)–Si(1)–C(1)–C(2)	12.8
Si(3)–C(4)	190.3				
Si(1)–C(5)	196.5				
C(1)–C(2)	135.9				
C(3)–C(4)	135.9				
C(5)–O(1)	123.5				

Figure S38. Calculated NPA charges of Me_4$^-$, Im1 and Im2.

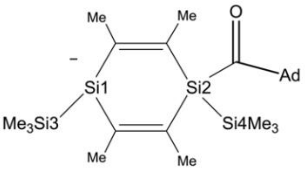

Me_4$^-$

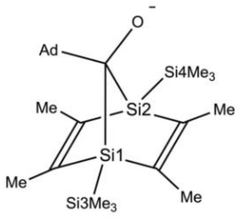

Im1

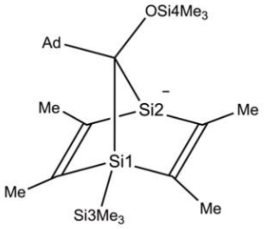

Im2

	Me_4$^-$	Im1	Im2
Si1	0.28	1.08	1.24
Si2	1.21	1.11	0.54
Si3	1.21	1.23	1.21
Si4	1.23	1.24	1.99
O	-0.63	-0.95	-0.97

Figure S39. UV absorption spectra of compounds **4** and **5** (n-hexane solution) including λ_{max} values (absorption coefficients in brackets)

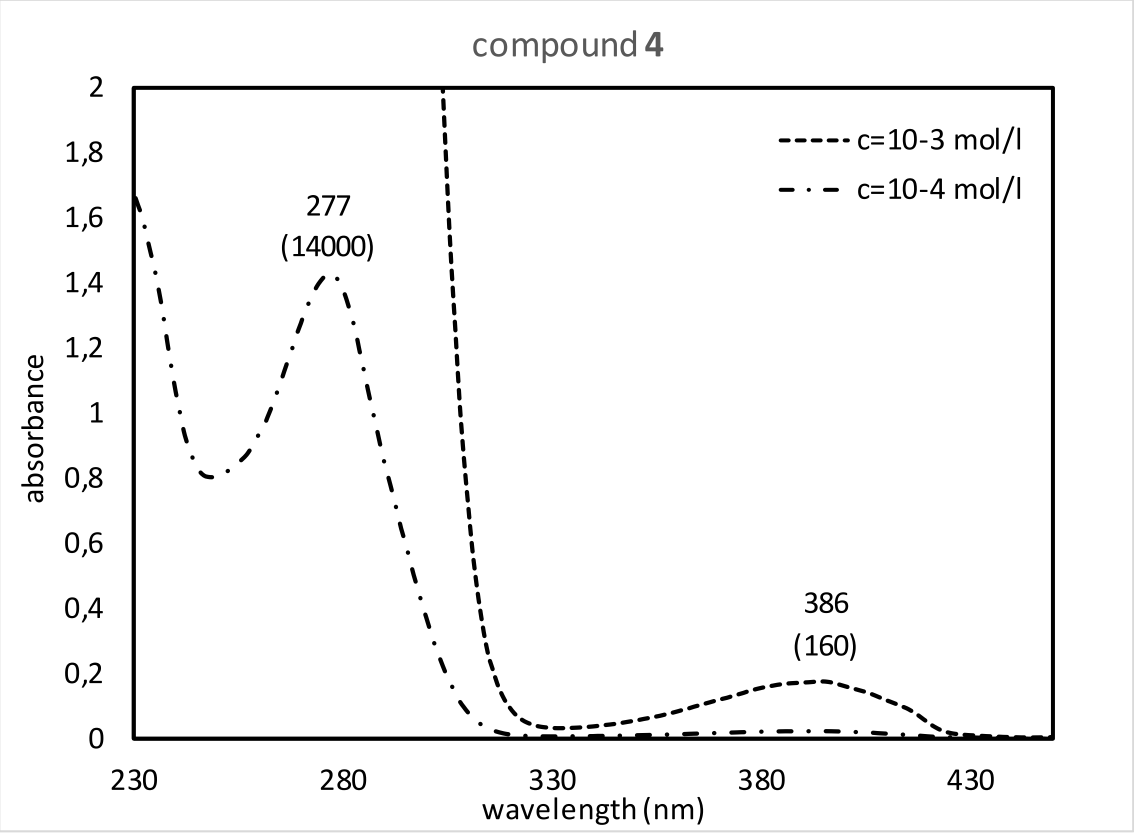

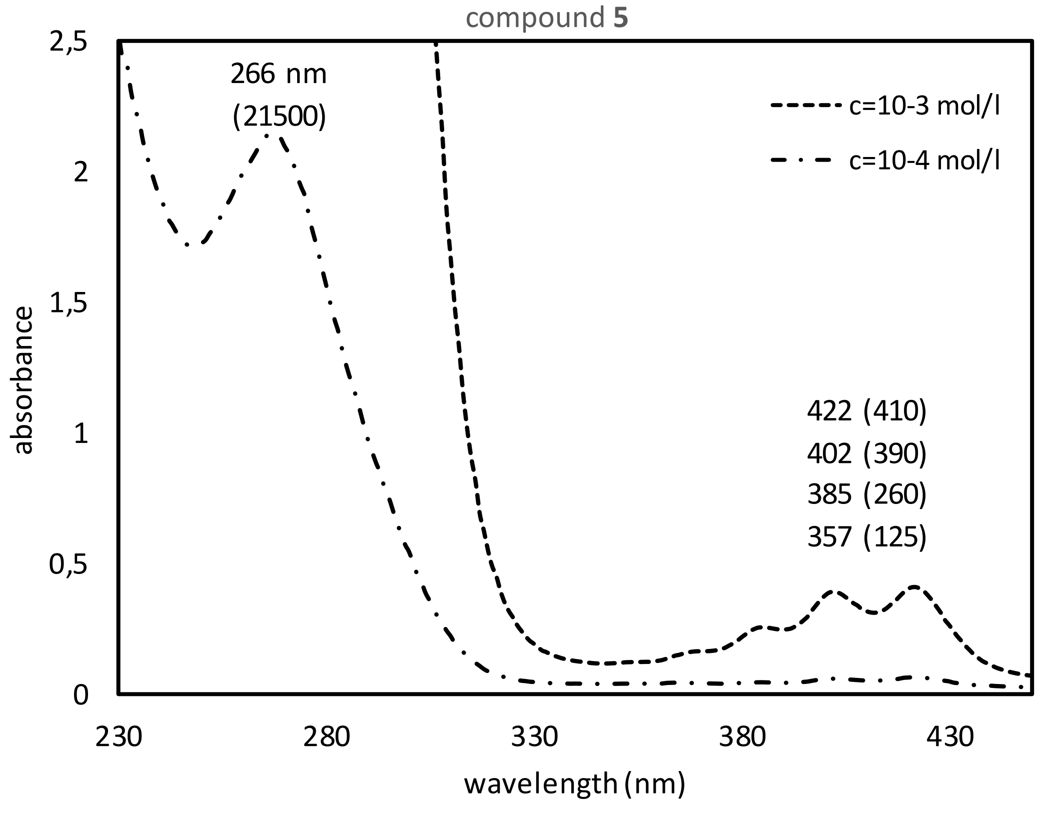

The UV absorption spectra of compounds **4** and **5** exhibit typical bands for acylsilanes and -germanes at 260 – 280 nm and in the visible range around 400nm.[1] These bands were assigned earlier to the $\pi \rightarrow \pi^*$ and $n \rightarrow \pi^*$ transitions of the Si-C(O)R. Unfortunately the former band superimposes the 273 nm band detected earlier in the absorption spectrum of 1,1,4,4-tetrakistrimethylsilyl-1,4-disilacyclo-hexa-2,5-diene **1** by Ottosson et al., which has been taken as an indicative for cross-hyperconjugative interactions in this molecule[2]. Concerning the study of cross-hyperconjugative interactions within **4** and **5**, thus, UV absorption spectroscopy apparently is not a suitable tool.

[1] a) P. C. Bulman Page, M. J. McKenzie, S. S. Klair, S Rosenthal In *The Chemistry of Organic Silicon Compounds*, Vol. 2; Rappoport, Z.; Apeloig, Y., Eds.; Wiley: Chichester, **1998**; p 1599. b) A. Eibel, J. Radebner, M. Haas, E. D. Fast, P. Faschauner, A. Torvisco, I. Lamparth, N. Moszner, H. Stueger, G. Gescheidt, *Polymer Chemistry* **2018**, *9*, 38. *DOI: 10.1039/C7PY01590A.*

[2] a) Tibbelin, J.; Wallner, A.; Emanuelsson, R.; Heijkenskjöld, F.; Rosenberg, M.; Yamazaki, K.; Nauroozi, D.; Karlsson, L.; Feifel, R.; Pettersson, R.; *et al.* 1,4-Disilacyclohexa-2,5-diene: a molecular building block that allows for remarkably strong neutral cyclic cross-hyperconjugation. *Chem. Sci.* **2014**, *5*, 360–371. b) Denisova, A. V.; Tibbelin, J.; Emanuelsson, R.; Ottosson, H. A Computational Investigation of the Substituent Effects on Geometric, Electronic, and Optical Properties of Siloles and 1,4-Disilacyclohexa-2,5-dienes. *Molecules* **2017**, *22*, 370.
